# A new nurse frog (Anura: *Allobates*) from Brazilian Amazonia with a remarkably fast multi-noted advertisement call

**DOI:** 10.7717/peerj.9979

**Published:** 2020-11-04

**Authors:** Jesus R.D. Souza, Miquéias Ferrão, James Hanken, Albertina P. Lima

**Affiliations:** 1 Programa de Pós-Graduação em Zoologia, Universidade Federal do Amazonas, Manaus, Amazonas, Brazil; 2 Departamento de Áreas Protegidas e Biodiversidade, Secretaria de Meio Ambiente do Acre, Rio Branco, Acre, Brazil; 3 Museum of Comparative Zoology, Harvard University, Cambridge, Massachusetts, USA; 4 Coordenação de Biodiversidade, Instituto Nacional de Pesquisas da Amazônia, Manaus, Amazonas, Brazil

**Keywords:** Advertisement call, Integrative taxonomy, Morphology, Phylogeny, Pulsed notes, State of Acre, Tadpole

## Abstract

Nurse frogs (Aromobatidae: *Allobates*) are probably the most extensively studied genus by taxonomists in Brazilian Amazonia. The southwestern portion of Amazonia is the most species-rich: as many as seven species may occur in sympatry at a single locality. In this study, we describe a new species of nurse frog from this region. The description integrates data from larval and adult morphology, advertisement calls and DNA sequences. *Allobates velocicantus* sp. nov. is distinguished from other *Allobates* mainly by the absence of hourglass-shaped dark marks on the dorsum and dark transverse bars on the thigh; a throat that is white centrally and yellow marginally; basal webbing on toes II and III; finger I longer than finger II; and an advertisement call composed of 66–138 pulsed notes with a note duration of 5–13 ms, inter-note intervals of 10–18 ms and a dominant frequency of 5,512–6,158 Hz. Tadpoles of the new species have 3–4 short, rounded papillae on the anterior labium, 16–23 papillae on the posterior labium, and a labial keratodont row formula 2(2)/3(1). This is the fifth species of *Allobates* described from the state of Acre, southwestern Brazilian Amazonia.

## Introduction

Nurse frogs of the genus *Allobates* Zimmermann & Zimmermann, 1988 inhabit the leaf-litter of Neotropical forests and are small and diurnal. They are distributed from northern Central America to the Atlantic Forest of Brazil (Grant et al., 2006, 2017; Frost, 2020). Currently, the genus comprises 55 nominal species (Frost, 2020), 22 of which are distributed in Brazilian Amazonia. Due to the general lack of substantial morphological differences among species, the integration of multiple independent lines of evidence (e.g., larval and adult morphology, vocalization, breeding behavior, and molecular data) have proven indispensable for the reliable discovery, diagnosis and formal description of new species (Lima et al., 2010; Lima, Simões & Kaefer, 2014, 2015; Simões, Lima & Farias, 2010; Simões et al., 2013; Simões, Rojas & Lima, 2019).

Because of its high degree of both species richness and hidden diversity, *Allobates* has attracted intense interest by systematists; *Allobates* may represent the most extensively studied genus of anurans in Brazilian Amazonia for the last two decades, a period in which 36 of the 55 species in the genus were described (Frost, 2020). Two to four species frequently cooccur in sympatry in several portions of Brazilian Amazonia, for example, Porto Walter (Grant et al., 2006; Melo-Sampaio, Oliveira & Prates, 2018) and Careiro (Lima et al., 2010; Lima, Ferrão & Silva, in press), in the states of Acre and Amazonas, respectively. However, southwestern Brazilian Amazonia harbors the highest level of species richness, with up to seven species sympatric at some localities, for example, the upper Madeira River (Dias-Terceiro et al., 2015). Six nominal species are known from the state of Acre (*A. femoralis* sensu lato, *A. flaviventris*, *A. gasconi*, *A. hodli, A. subfolionidificans* and *A. trilineatus* sensu lato: Lima, Sanchez & Souza, 2007; Simões, Lima & Farias, 2010; Melo-Sampaio, Souza & Peloso, 2013; Melo-Sampaio, Oliveira & Prates, 2018) and several other candidate species await formal description (Grant et al., 2006; Melo-Sampaio, Souza & Peloso, 2013; Grant et al., 2017; Melo-Sampaio, Oliveira & Prates, 2018).

During a herpetological survey of forests along federal highway BR364 and vicinal roads in the state of Acre, A. P. Lima found a species of *Allobates* with a long-lasting call consisting of rapidly emitted notes. We compared this call, as well as the morphology and mtDNA of these specimens, to other described species and concluded that they belong to a yet unnamed species. In the present study, we describe this new species through integrative taxonomy. In addition, we describe its breeding behavior, from courtship to tadpole emergence and transport.

## Materials and Methods

**Sampling.** Adult specimens were collected 13–14 January 2019 along state highway AC405 (7°38′54″ S, 72°48′59″ W, 211 m a.s.l.), municipality of Mâncio Lima, state of Acre, Brazil. Also collected from this locality were a group of tadpoles obtained from the back of INPAH 41347 (field number APL 21403), an adult male, and a clutch of fertilized eggs obtained soon after oviposition. In addition, an adult male was collected 15 February 2019 along the road (7°34′02″ S, 72°39′32″ W, 181 m a.s.l) connecting the municipality of Cruzeiro do Sul (state of Acre) to Guajará (state of Amazonas). Adults were euthanized with a 2% benzocaine topical solution, fixed in 10% neutral-buffered formalin and preserved in 70% ethanol. Tissue samples were obtained before immersion in formalin. Tadpoles and eggs were reared in the laboratory until they reached Gosner stages 27–37 for morphological description, when they were euthanized with a 10% aqueous benzocaine solution and preserved in 5% neutral-buffered formalin. Adults and tadpoles were housed in the herpetological section of the Zoological Collection of the Instituto Nacional de Pesquisas da Amazônia (INPAH), Manaus, Amazonas, Brazil. Protocols of collection and animal care follow the Conselho Federal de Biologia resolution number 148/2012 (Conselho Federal de Biologia–CFB, 2012). Specimens were collected under collection permit number 1337-1 provided by the Instituto Brasileiro do Meio Ambiente e dos Recursos Naturais Renováveis (IBAMA).

The advertisement calls of eight males were recorded. Seven males were recorded in the state of Acre: six along highway AC405 (7°38′54″ S, 72°48′59″ W, 211 m a.s.l.) and one along the road connecting the municipality of Cruzeiro do Sul–Guajará (7°34′02″ S, 72°39′32″ W, 181 m a.s.l). The eighth male was recorded near the municipality of Guajará (7°27′10″ S, 72°35′14″ W, 225 m a.s.l.), Amazonas, Brazil. Recordings were made between 06:30 h and 11:00 h and between 15:00 h and 17:30 h. Air temperature at the time of recording, measured with a digital thermohygrometer positioned 1 m above the ground, ranged from 24 to 27.4 °C. Calls were recorded with a directional microphone Shotgun CSR Yoga HT-81 accoupled to a Zoom H4n digital recorder. The microphone was positioned approximately 1 m in front of the focal active male. Recordings were stored with 16 bits resolution, 44.1 kHz and WAV format. Call recordings were deposited in the Fonoteca Neotropical Jacques Vielliard (FNJV 45469–79), UNICAMP, Campinas, Brazil.

**Sequencing and phylogenetic analysis.** Total genomic DNA was extracted from tissue samples of five specimens of the new species using the commercial kit Wizard (Promega Corp., Madison, WI, USA) following the manufacturer’s instructions. Fragments of the 16S rRNA mitochondrial gene were amplified by polymerase chain reaction (PCR) using universal primers 16sar (5′-CGCCTGTTTATCAAAAACAT-3′) and 16sbr (5′-CCGGTCTGAACTCAGATCACGT-3′) (Palumbi, 1996). Extractions, PCR reactions and sequencing were conducted with both forward and reverse primers (Maia, Lima & Kaefer, 2017). Sequences were visually checked and manually edited in Geneious *5.3.4* (Kearse et al., 2012). Sequences were deposited at GenBank and can be accessed under numbers MT446458–62.

We used BLAST to search for sequences already deposited in GenBank that might correspond to the new species. Two 16S rRNA sequences, KY886579 and MF624181, matched our sequences with high identity. Additionally, we retrieved 68 additional 16S sequences that represent the 37 species of *Allobates* currently available in GenBank (Table S1). Sequences of four species of other genera in the family Aromobatidae were used to root the tree: *Anomaloglossus stepheni* (Martins, 1989), *Aromobates nocturnus* Myers, Paolillo & Daly, 1991, *Mannophryne collaris* (Boulenger, 1912) and *Rheobates palmatus* (Werner, 1899). For phylogenetic reconstruction, we downloaded additional sequences of three other mitochondrial genes (12S ribosomal RNA; *cytochrome oxidase I*, COI; and *cytochrome b*, cytb) and six nuclear genes (28S *ribosomal* RNA, 28S; *histone H3*, HH3; *recombination activating gene 1*, RAG1; *rhodopsin*, RHO; *seventh* in absentia, SIA; and *tyrosinase*, TYR) from the same specimens (when available) for which 16S sequences were initially downloaded. Sequences were aligned in Bioedit 7.*2.5* (Hall, 1999) using the CLUSTAL W algorithm (Thompson, Higgins & Gibson, 1994). Alignments were concatenated in Mesquite 3.04 (Maddison & Maddison, 2015), which yielded a final alignment consisting of 5,915 base pairs (bp) and 79 terminals. Vouchers and GenBank accession numbers are listed in Table S1.

The best-fit partition scheme and most probable nucleotide evolution model, considering codon partitioning for protein-coding genes, were inferred with PartitionFinder 2.1.1 (Lanfear et al., 2016) via the CIPRES webserver (phylo.org) using Bayesian Inference Criterion and the PhyML algorithm (Guindon et al., 2010). The best-fit partition schemes and evolution models for each partition are shown in Table 1. Phylogenetic relationships were reconstructed using Maximum Likelihood inference in IQ-TREE (Trifinopoulos et al., 2016). Clade support was calculated through 10,000 ultrafast bootstrap approximation replicates with 10,000 maximum iterations, a 0.99 minimum correlation coefficient and 10,000 replicates of the Shimodaira-Hasegawa approximate likelihood ratio. Kimura 2-parameter distance (Kimura, 1980) and uncorrected genetic distance were calculated using the 16S mitochondrial gene through *MEGA 6.0* (Tamura et al., 2013).

**Table 1:** Selected partitions and evolutionary models proposed by Bayesian Inference Criterion through the PhyML algorithm.

PT	Model	Genes	Position of base pairs
1	GTR+I+G	12S 16S	1–946 5238–5915
2	JC+I	28S HH3\2 HH3\3 SAI\1 SAI\2	947–1741 3185–3511\3 3186–3511\3 4264–4660\3 4265–4660\3
3	GTR+I+G	COI\1 CYTB\1	1742–2339 2340–3183\3
4	SYM+I+G	COI\2 CYTB\2 CYTB\3	1743–2339\3 2341–3183\3 2342–3183\3
5	F81	COI\3 RHO\3	1744–2339\3 3949–4263\3
6	HKY+G	HH3\1 RHO\1 TYR\3	3184–3511\3 3947–4263\3 4663–5237\3
8	K80+G	RAG1\1 SAI\3	3512–3946\3 4266–4660\3
9	HKY+I	RAG1\2 RAG1\3 RHO\2 TYR\1 TYR\2	3513–3946\3 3514–3946\3 3948–4263\3 4661–5237\3 4662–5237\3

**Note:**

Numbers after gene names denote the codon position. PT, partitions.

**Morphology.** Sex was determined by direct observation of secondary sexual characters (e.g., vocal sac and vocal slits in males) and breeding behavior (advertisement call by males, females surrounding active males). Morphometric measurements longer than 10 mm were taken using a digital caliper (to the nearest 0.1 mm); shorter measurements were taken with a micrometer coupled to a stereoscopic microscope. Measurements follow Lima, Sanchez & Souza (2007) and Barrio-Amorós & Santos (2009): snout-vent length (SVL); head length (HL); interorbital distance (IO); head width (HW); snout length (SL); eye-nostril distance (END); inter-nostril distance (IND); eye length (EL); horizontal tympanum diameter (TYM); forearm length (FAL); upper arm length (UAL); thigh length (LL); tibial length (TL); foot length (FL); hand length from the proximal edge of the palmar tubercle to the tip of finger I (HANDI), finger II (HANDII), finger III (HANDIII), and finger IV (HANDIV); disc width of finger III (WFD); palmar tubercle diameter (DPT); width of thenar tubercle (WTT); width of finger III at proximal phalanx (WFP); width of toe IV disc (WTD). Morphological terminology follows Grant et al. (2006). The description and diagnosis follow Lima, Simões & Kaefer (2014). See Table S2 for morphometric measurements of the type series.

Developmental stages of tadpoles (lot INPAH 41351) were determined following Gosner (1960). Terminology, diagnostic features, and measurements follow Altig & McDiarmid (1999) and Schulze, Jansen & Köhler (2015). Tadpole descriptions follow Schulze, Jansen & Köhler (2015). The following morphometric measurements were taken with a micrometer coupled to a stereoscopic microscope: total length (TL), measured from tail tip to snout tip; body length (BL), measured from snout tip to tail insertion; tail length (TAL), measured from tail tip to its insertion into the body; body width (BW); body height (BH); head width (HWLE); tail muscle width (TMW); maximum tail height (MTH); tail muscle height (TMH); interorbital distance (IOD); inter-nostril distance (IND); eye-nostril distance (END); nostril-snout distance (NSD); eye diameter (ED); vent tube length (VTL); spiracle tube length (STL); and oral disc width (ODW).

**Call description.** Parameters were measured using Raven 1.5 (Bioacoustics Research Program, 2015). The following parameters were measured: call duration (CD), inter-call interval (ICI), notes per calls (NNC), note duration (ND), inter-note interval (INI), note repetition rate (NRR), call dominant frequency (DF), and first-note dominant frequency (FNDF). We analyzed 1–5 calls for each recorded male and 10 notes and their respective inter-note intervals in each call. Measured notes were equally distributed along each call. Bioacoustic terminology follows Köhler et al. (2017). Spectral parameters were measured by spectrograms set as Window = Blackman; Discrete Fourier Transform = 2,048 samples; 3 dB filter bandwidth = 82 Hz. Graphic representation of the advertisement calls was generated in the R environment (R Core Team, 2016) using the Seewave package 2.0.5 (Sueur, Aubin & Simonis, 2008). Seewave was set up as follows: Hanning window, overlap of 85%, and 256 points of resolution (FFT).

**Breeding behavior.** Notes regarding breeding behavior were taken from a pair of frogs observed between 07:00 and 08:20 h on 14 February 2019 along state highway AC405 (7°38′54″ S, 72°48′59″ W, 211 m a.s.l.), municipality of Mâncio Lima, state of Acre, Brazil. A male (INPAH 41347) was observed carrying tadpoles on his back at the same locality later that day.

**Figure 1:**
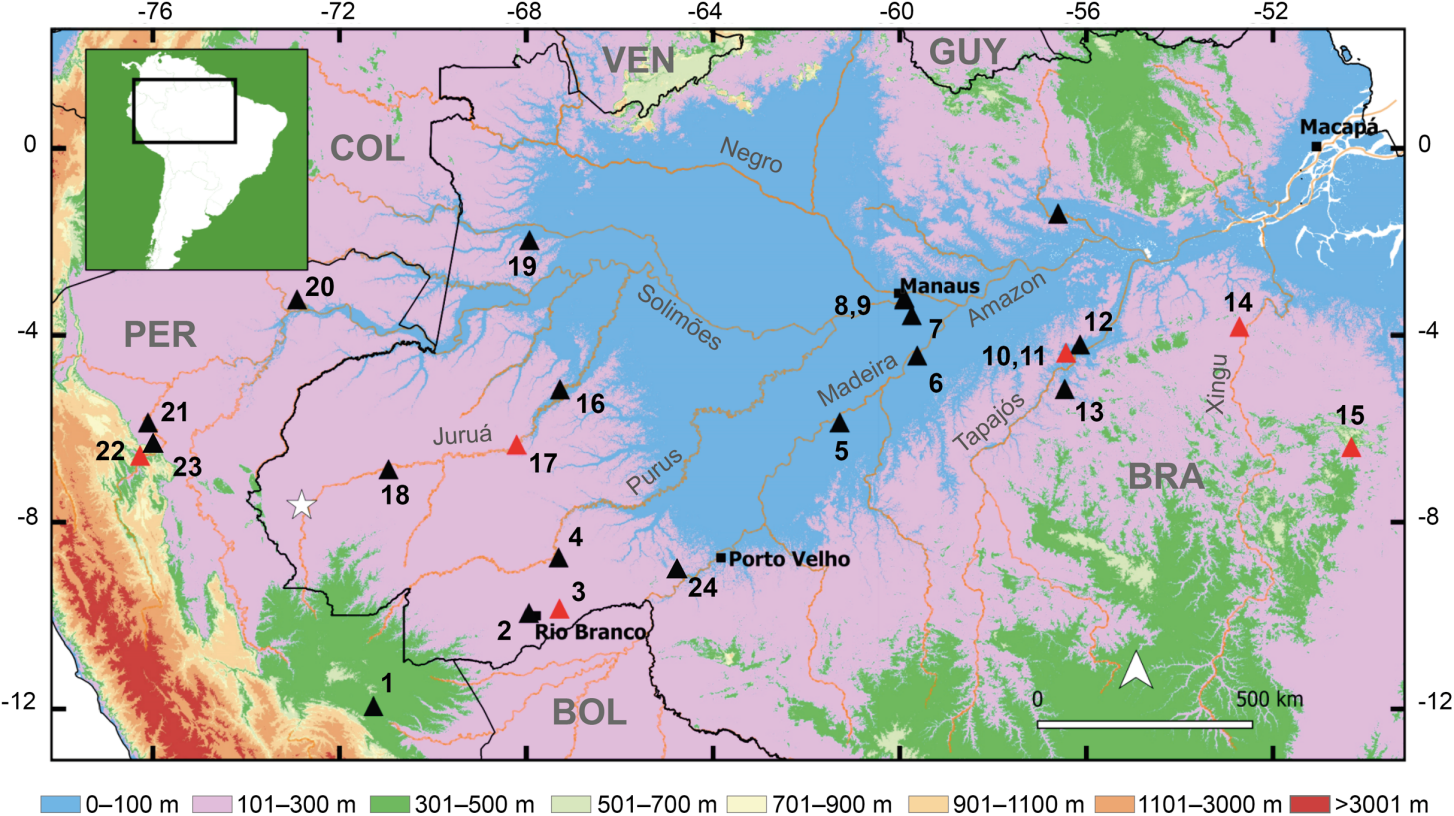
Geographic distribution of the new species and the type localities of other cryptically colored *Allobates* to which it is compared. White star denotes the type locality of the new species. Triangles and numbers denote the type localities of other species: (1) *A. conspicuus*; (2) *A. subfolionidificans*; (3) *A. flaviventris* (4) *A. tinae*; (5) *A. bacurau*; (6) *A. grillisimilis*; (7) *A. nidicola*; (8) *A. palevarzensis*; (9) *A. caeruleodactylus*; (10) *A. magnussoni*; (11) *A. tapajos*; (12) *A. masniger*; (13) *A. nunciatus*; (14) *A. crombiei*; (15) *A. carajas*; (16) *A. vanzolinius*; (17) *A. gasconi*; (18) *A. fuscellus*; (19) *A. juami*; (20) *A. melanolaemus*; (21) *A. trilineatus*; (22) *A. ornatus*; (23) *A. femoralis*; (24) *A. hodli*. Red triangles denote species with dark marks on the dorsum. Abbreviations: BOL, Bolivia; BRA, Brazil; COL, Colombia; GUY, Guyana; PER, Peru; VEN, Venezuela.

**Interspecific comparisons.** The new species inhabits lowland forest in southwestern Brazilian Amazonia near the border with Peru. Hence, we compare the new species with congeners distributed in Brazilian Amazonia south of the Amazon River and in Bolivian and Peruvian Amazonia (Fig. 1): *Allobates bacurau* Simões, 2016; *A. brunneus* (Cope, 1887), *A. caeruleodactylus* (Lima & Caldwell, 2001); *A. carajas* Simões, Rojas & Lima, 2019; *A*. *conspicuus* (Morales, 2002); *A. crombiei* (Morales, 2002); *A. femoralis* (Boulenger, 1884), A. *flaviventris* Melo-Sampaio, Souza & Peloso, 2013; *A. fuscellus* (Morales, 2002); *A. gasconi* (Morales, 2002); *A. grillisimilis* Simões et al., 2013; *A. hodli* Simões, Lima & Farias, 2010; *A. magnussoni* Lima, Simões & Kaefer, 2014; *A. masniger* (Morales, 2002); *A. melanolaemus* (Grant & Rodríguez, 2001); *A. nidicola* (Caldwell & Lima, 2003); *A. nunciatus* Moraes, Pavan & Lima, 2019; *A*. *ornatus* (Morales, 2002); *A. pacaas* Melo-Sampaio et al., 2020; *A. paleovarzensis* Lima et al., 2010; *A. subfolionidificans* (Lima, Sanchez & Souza, 2007); *A. tapajos* Lima, Simões & Kaefer, 2015; *A. tinae* Melo-Sampaio, Oliveira & Prates, 2018; *A*. *trilineatus* (Boulenger, 1884); and *A. vanzolinius* (Morales, 2002). In addition, we compare the new species to *A. insperatus* (Morales, 2002) and *A. juami* Simões et al., 2018, which have similar calls. In the following description, characters of compared species are presented inside parentheses unless stated otherwise.

**Nomenclatural acts.** The electronic version of this article in Portable Document Format (PDF) will represent a published work according to the International Commission on Zoological Nomenclature (ICZN), hence the new names contained in the electronic version are effectively published under that Code from the electronic edition alone. This published work and the nomenclatural acts it contains have been registered in ZooBank, the online registration system for the ICZN. The ZooBank LSIDs (Life Science Identifiers) can be resolved and the associated information viewed through any standard web browser by appending the LSID to the prefix http://zoobank.org/. The LSID for this publication is: urn:lsid:zoobank.org:pub:B411120A-AB6C-4091-81D7-24D52B377BEE. The online version of this work is archived and available from the following digital repositories: PeerJ, PubMed Central and CLOCKSS.

## Results

**Figure 2:**
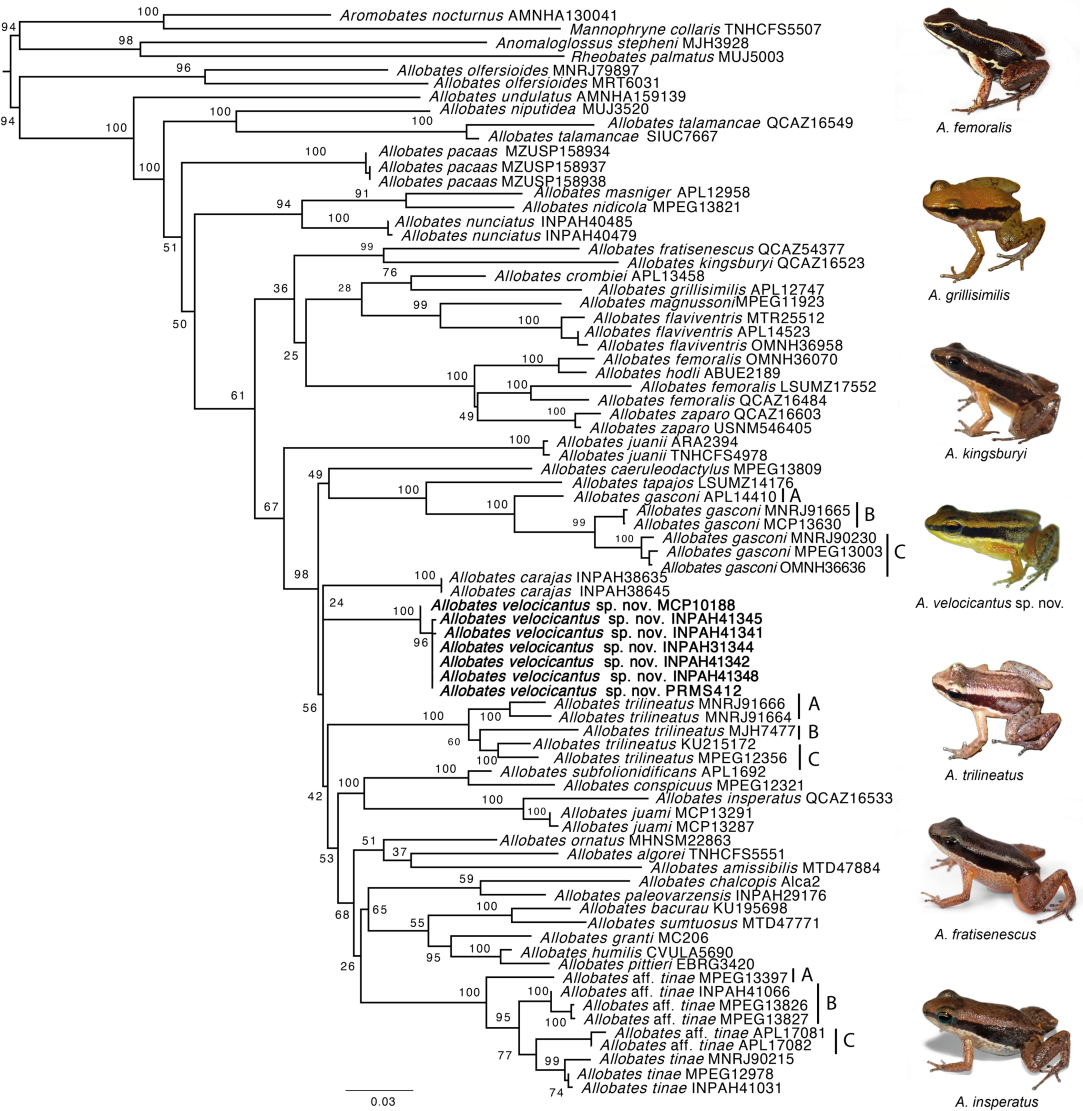
Phylogeny of the genus *Allobates* reconstructed based on Maximum Likelihood analysis of four mitochondrial genes (12S, 16S, COI, CYTB) and six nuclear genes (28S, HH3, RAG1, RHO, SIA, TYR). Clade support from 10,000 bootstraps are depicted close to nodes. Photographs by Santiago Ron—www.bioweb.bio (*A. insperatus*, *A. femoralis*, *A. fratisenescus*, *A. trilineatus*, and *A. kingsburyi*), and Jesus R. D. Souza (*A. velocicantus* sp. nov.).

Our phylogeny is mostly consistent with previously published phylogenetic hypotheses (Grant et al., 2006; Grant et al., 2017; Melo-Sampaio, Oliveira & Prates, 2018) (Fig. 2). The monophyly of *Allobates* is highly supported (ML = 94%), with *A. olfersioides* emerging as the sister group to all other congeners (ML = 100%). It is followed by *A. undulatus* (ML = 100%) and a clade formed by *A. niputidea* and *A. talamancae* (ML = 100%). Despite the differences between the phylogenetic relationships of some clades recovered in this study and those of previously published phylogenies, most known clades are recovered in our analyses, such as the one composed of *A. nunciatus*, *A. nidicola* and *A. masniger*, and the one that groups *A. subfolionidificans*, *A. conspicuous*, *A. insperatus* and *A. juami* (Fig. 2).

Samples of the new species nest with two other GenBank samples from Cruzeiro do Sul (Acre, Brazil), MF624181 and KY886579, which together form a well-supported clade (ML = 100%). While *Allobates carajas* is retrieved as sister to the new species, support for this relationship is very weak (ML = 24%). Although they share a similar call structure, the new species is not closely related to *A. crombiei*, *A. amissibilis* or *A. juami*. *Allobates insperatus* and *A. juami* are recovered as sister species with strong support (ML = 100%), whereas *A. crombiei* is sister to *A. grillisimilis* with weak support (ML = 76%).

The average uncorrected pairwise distance (*p*-distance) between the new species and its cryptically colored congeners is large: 9.6% ± 1.5 (Table 2). The smallest *p*-distance is to *Allobates trilineatus* clade A (6.6%) and the largest is to *A. olfersioides* (13.5%). Distances between the new species and *A. insperatus* (9.7%), *A. juami* (9.5%) and *A. crombiei* (9.9%) are similar to or larger than the average distance between the new species and the entire dataset. See Table 2 for interspecific K2P distances. Average intraspecific genetic distances are small (*p*-distance and K2P = 0.3%).

**Table 2:** Pairwise interspecific genetic distances between *Allobates velocicantus* sp. nov. and other species of *Allobates* included in the phylogenetic tree.

Species	*p*	K2P	Species	*p*	K2P
*Allobates algorei*	9.7	10.5	*Allobates masniger*	10.8	11.8
*Allobates amissibilis*	8.9	9.6	*Allobates nidicola*	10.4	11.4
*Allobates bacurau*	10.0	10.8	*Allobates niputidea*	11.4	12.6
*Allobates caeruleodactylus*	7.7	8.2	*Allobates nunciatus*	9.6	10.4
*Allobates carajas*	6.8	7.2	*Allobates olfersioides*	13.5	15.0
*Allobates chalcopis*	8.2	8.7	*Allobates ornatus*	9.5	10.3
*Allobates conspicuus*	9.5	10.3	*Allobates pacaas*	10.3	11.2
*Allobates crombiei*	9.9	10.7	*Allobates paleovarzensis*	7.5	7.9
*Allobates flaviventris*	10.0	10.8	*Allobates pittieri*	11.8	13.1
*Allobates fratisenescus*	9.4	10.1	*Allobates subfolionidificans*	9.9	10.7
*Allobates gasconi* A	9.1	9.8	*Allobates sumtuosus*	8.9	9.5
*Allobates gasconi* B	9.2	9.9	*Allobates talamancae*	11.2	12.3
*Allobates gasconi* C	9.7	10.4	*Allobates tapajos*	9.8	10.7
*Allobates granti*	12.1	13.5	*Allobates tinae*	9.4	10.1
*Allobates grillisimilis*	9.9	10.8	*Allobates* aff. *tinae* A	9.0	9.7
*Allobates humilis*	10.2	11.1	*Allobates* aff. *tinae* B	9.2	9.9
*Allobates insperatus*	9.7	10.5	*Allobates* aff. *tinae* C	8.9	9.5
*Allobates juami*	9.5	10.3	*Allobates trilineatus* A	6.8	7.2
*Allobates juanii*	11.2	12.2	*Allobates trilineatus* B	6.6	6.9
*Allobates kingsburyi*	8.8	9.5	*Allobates trilineatus* C	6.8	7.2
*Allobates magnussoni*	9.7	10.5	*Allobates undulatus*	11.9	13.1

**Note:**

Genetic distances, presented as percentages, were calculated based on a 678-bp fragment of the 16s rRNA gene. Abbreviations: *p*, *p*-distance; K2P, Kimura 2-parameter distance.

## Taxonomy

*Allobates velocicantus* **sp. nov**.

LSID: urn:lsid:zoobank.org:act:8EAF9324-F176-4A5F-8788-9ACE3E885173.

*Allobates* sp. CdS MCP10187 (MCP10188) Grant et al. (2017).

**Holotype.** INPAH 41342 (field number APL 21398, GenBank accession number MT446461), an adult male from along state highway AC405, municipality of Mâncio Lima, state of Acre, Brazil (7°38′54″, 72°48′59″ W, 211 m a.s.l.), collected by J.R.D. Souza and A.P. Lima on 13 January 2019.

**Paratopotypes.** Eleven specimens: eight males, INPAH 41338–40 (field numbers APL 21391–93), 41341(field number APL 21397, GenBank accession number MT446462), 41343 (field number APL 21399), 41346–47 (field numbers APL 21402–03), 41348 (field number APL 21404, GenBank accession number MT446458); and three females, INPAH 41344–45 (field numbers APL 21400 (GenBank accession number MT446460) and 21401 (GenBank accession number MT446459)), and 41349 (field number APL 21405); collected by J.R.D. Souza and A.P. Lima on 13 and 14 January 2019. INPAH 413445 is the designated allotype.

**Paratype.** One adult male, INPAH 41350 (field number APL 21410), from along the road connecting Cruzeiro do Sul to Guajará (7°34′2″ S; 72°39′32″ W, 181 m a.s.l.), municipality of Cruzeiro do Sul, state of Acre, Brazil, collected by J.R.D. Souza on 15 February 2019.

**Etymology.** The specific epithet is derived from the Latin words *velox* (= fast) and *cantus* (= singing), in reference to the high note-repetition rate of the advertisement call of the new species. Proposed standard English name: fast singer frog. Proposed standard Spanish name: sapito del canto veloz. Proposed standard Portuguese name: sapinho do canto acelerado.

**Phylogenetic placement.** The new species is assigned to the genus *Allobates* based on its phylogenetic position presented in the present study and in Grant et al. (2017).

**Diagnosis**. *Allobates velocicantus* sp. nov. is characterized by (1) small size, SVL 14.9–16.2 mm in males and 16.0–17.4 mm in females; (2) dorsal color pattern predominantly light brown, with no dark patches and marks; (3) dorsum granular; (4) dark brown lateral stripe from the tip of the snout to the groin; (5) light dorsolateral stripe absent or inconspicuous in living specimens but present in preserved specimens; (6) light but incomplete ventrolateral stripe; (7) oblique lateral line diffuse; (8) snout slightly rounded in dorsal view; (9) tympanum inconspicuous; (10) paired dorsal digital scutes; (11) supernumerary tubercles absent; (12) distal tubercle absent on finger IV; (13) discs moderately expanded on fingers I–IV; (14) finger I slightly larger than finger II; (15) finger III of males with similar width along phalanges; (16) dermal lateral fringes and webbing absent on fingers; (17) metacarpal ridge absent; (18) carpal pad absent; (19) excrescences on thumbs absent; (20) dark gland on arm absent; (21) basal webbing present between toes III and IV; (22) metatarsal tubercle present; (23) light “half-moon” shaped paracloacal mark; (24) superior eyelids dark brown; (25) iris metallic bronze; (26) pupil large, black, horizontal semielliptical; (27) transverse bars on thigh absent; (28) dark brown spots on tibia; and (29) advertisement calls formed by 66–138 pulsed notes (emitted in multiple exhalations) with a dominant frequency of 5,512–6,158 Hz.

**Figure 3:**
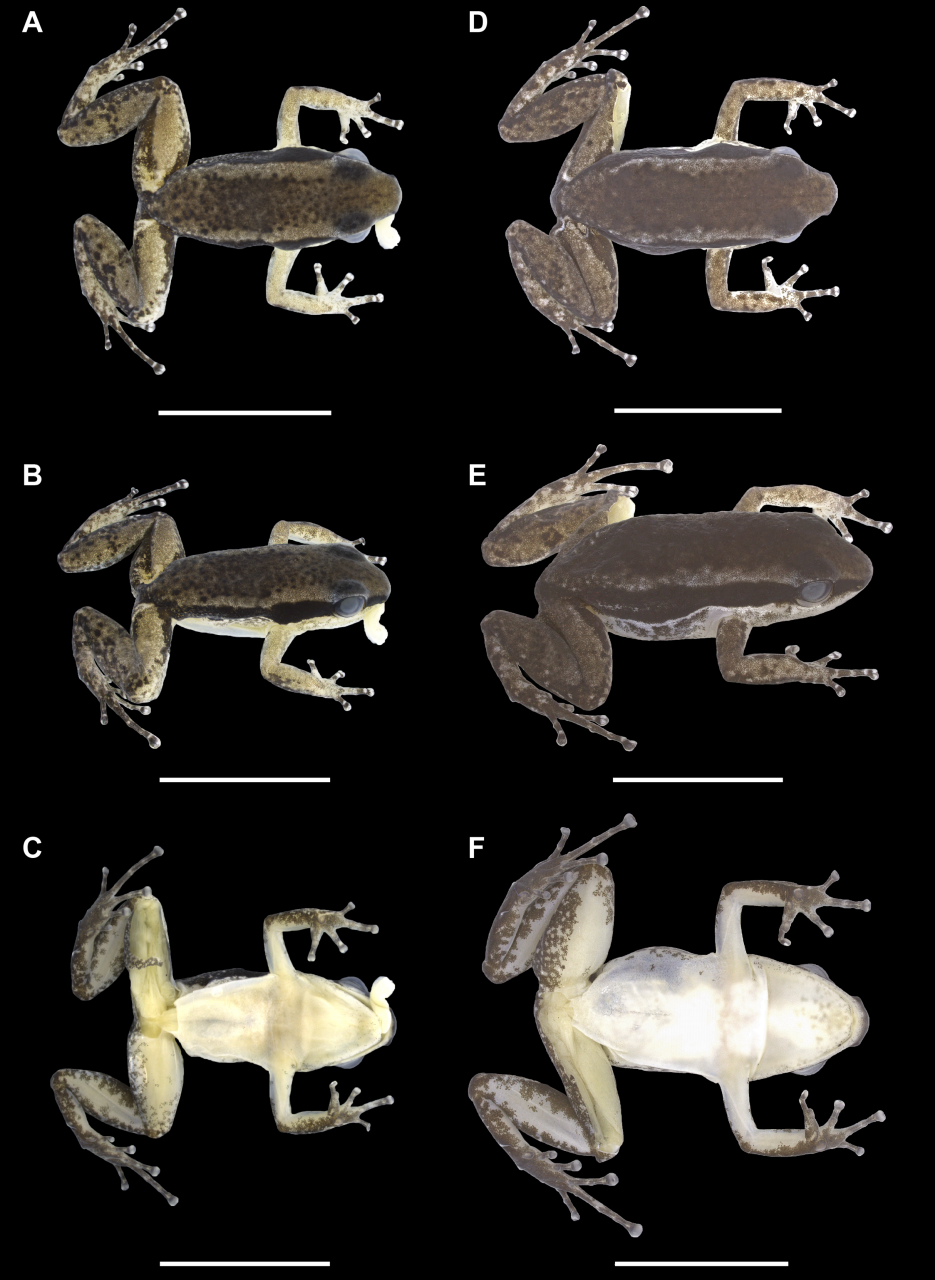
Dorsal, dorsolateral and ventral views of the holotype (INPAH 41342) and allotype (INPAH 41345) of *Allobates velocicantus* sp. nov. (A–C) Holotype. (D–F) Allotype. Scale bar: 10 mm. Photographs by Jeni Lima Magnusson.

**Figure 4:**
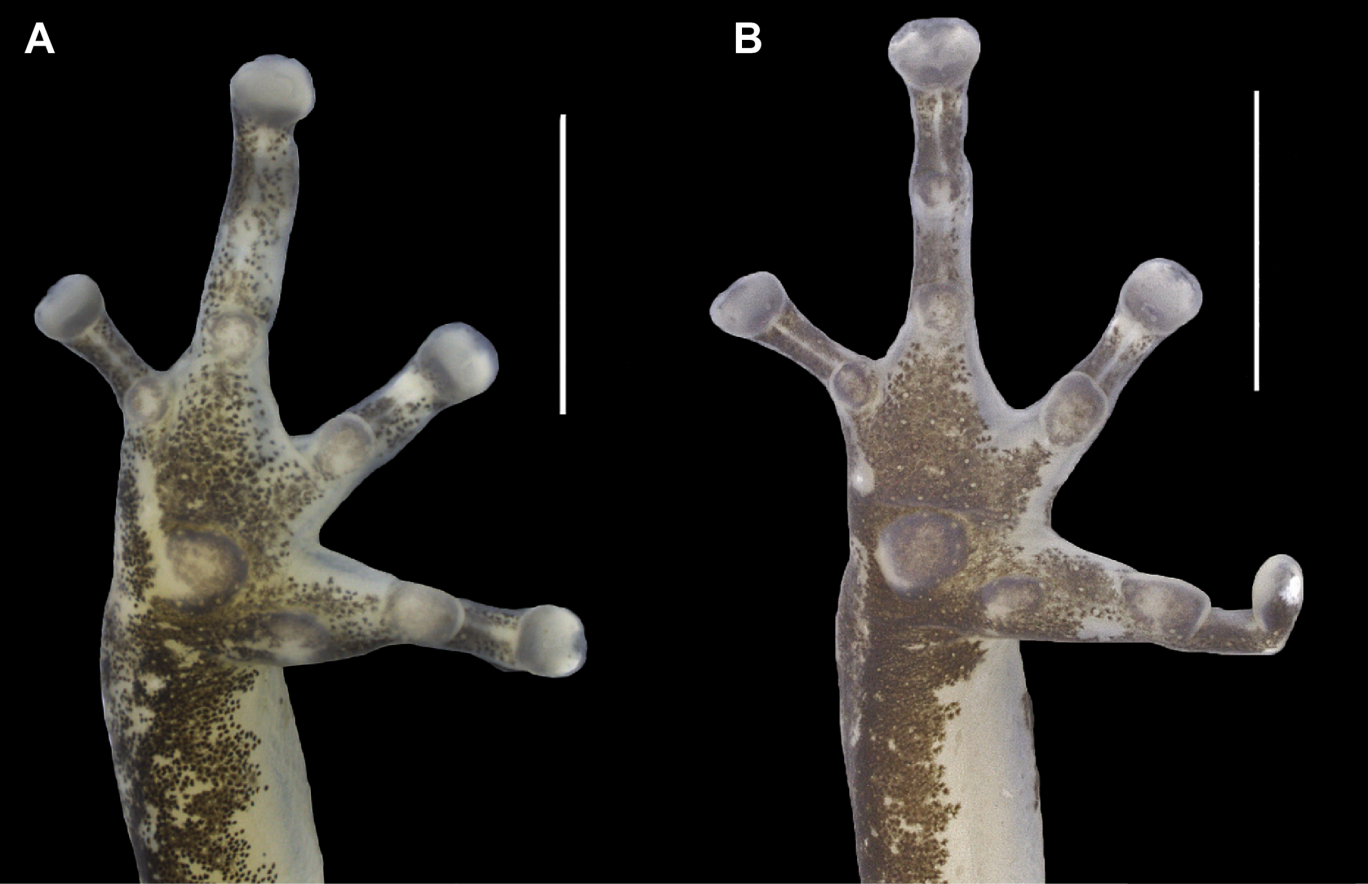
Ventral view of the right hand of the holotype (INPAH 41342) and allotype (INPAH 41345) of *Allobates velocicantus* sp. nov. (A) Holotype. (B) Allotype. Scale bar: 2 mm. Photographs by Jeni Lima Magnusson.

**Figure 5:**
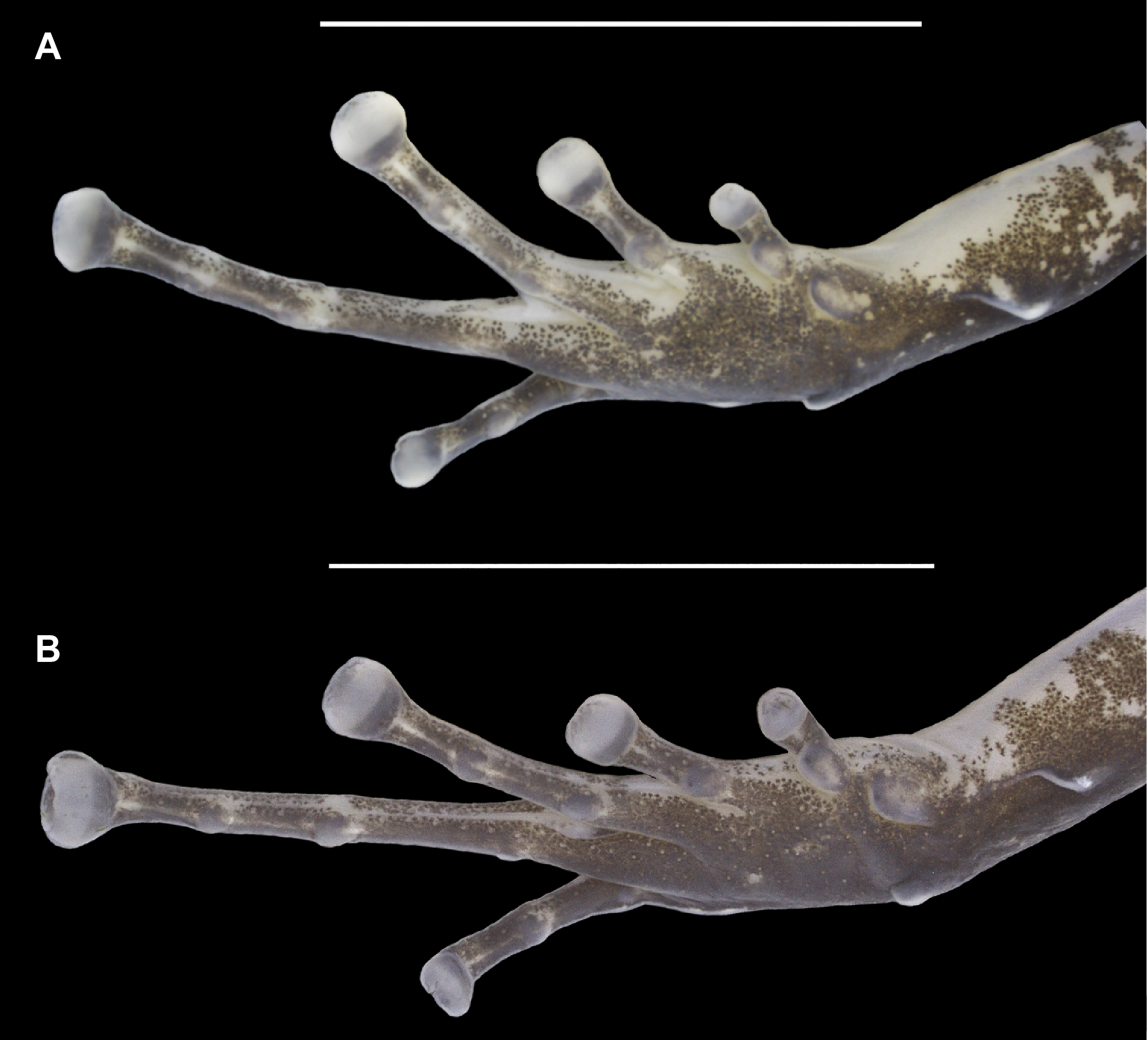
Ventral view of the right foot of the holotype (INPAH 41342) and allotype (INPAH 41345) of *Allobates velocicantus* sp. nov. (A) Holotype. (B) Allotype. Scale bar: 5 mm. Photographs by Jeni Lima Magnusson.

**Figure 6:**
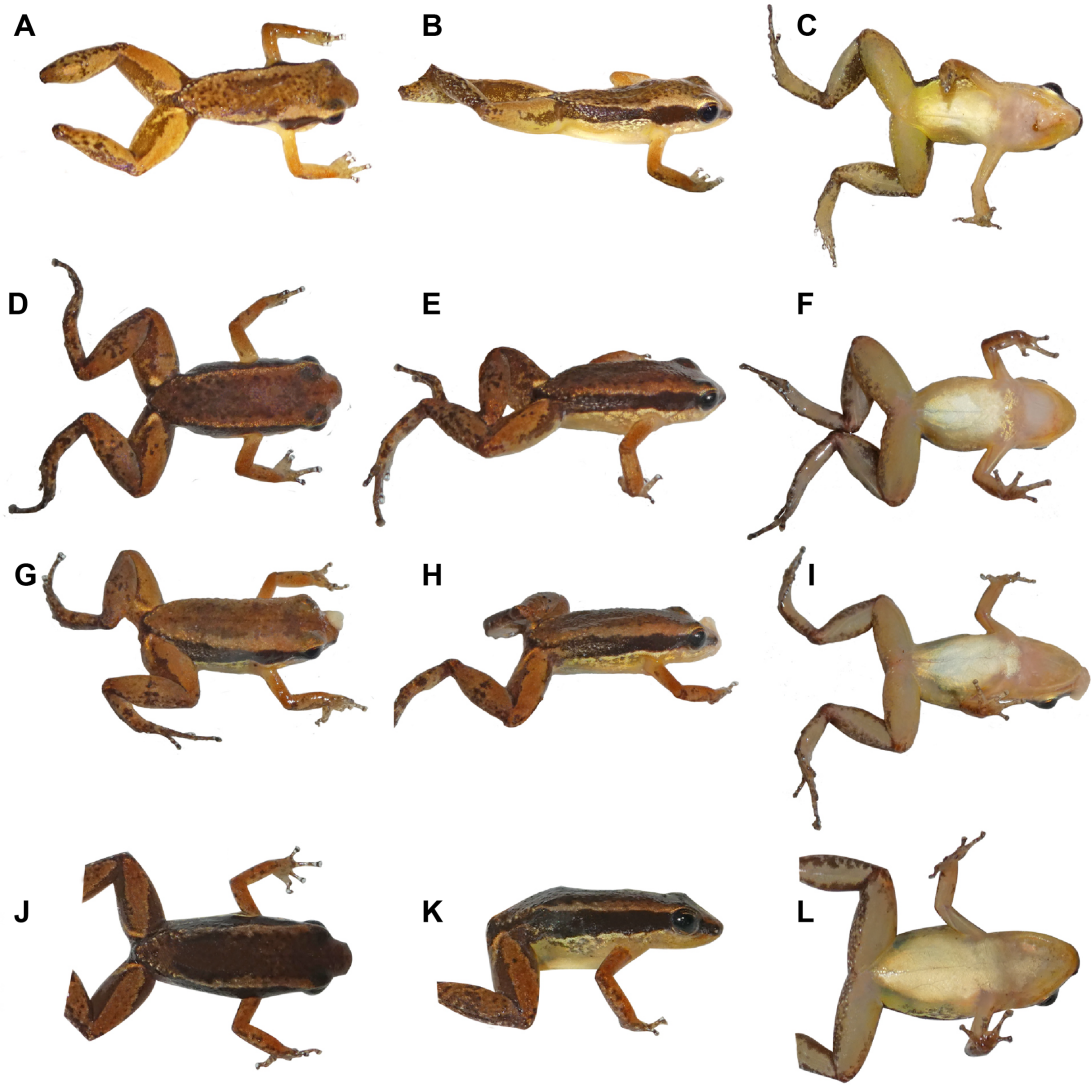
Coloration in life of adult *Allobates velocicantus* sp. nov. (A–C) Holotype, male, INPAH 41342, SVL = 15.3 mm. (D– F) Male, INPAH 41341, SVL = 15.4 mm. (G–I) Female, INPAH 41344, SVL = 16.6 mm. (J–L) Female, INPAH 41345, SVL = 17.4 mm. Photographs by Albertina Pimentel Lima.

**Description of the holotype.** Adult male, INPAH 41342 (field number APL 21398), SVL = 15.3 mm (Figs. 3A–3C, 4A, 5A and 6A–6C). Measurements of the holotype are presented in Table 3. Head 23% wider than long. Head width and length equal 35% and 30% of SVL, respectively. Eye diameter larger than distance from eye to nostril (EN/EL = 0.59). Eye diameter 50% of head length. Interorbital distance 81% of head width. Tympanum inconspicuous to the naked eye. Snout rounded in dorsal and lateral views. Nares located laterally at the tip of the snout. *Canthus rostralis* barely defined, slightly straight in dorsal view and rounded in lateral view; loreal region slightly concave. Maxillary teeth absent. Median lingual process absent. Tongue three times longer than wide (3.6 mm vs. 1.2 mm). Vocal sac single and subgular. Lateral vocal slits present at the height of the jaw angle.

Palmar tubercle rounded, conspicuous, 0.6 mm wide. Thenar tubercle elliptical, conspicuous, 0.25 mm wide. Thenar tubercle width 42% of palmar tubercle diameter. Distal subarticular tubercle on finger III laterally positioned, tiny and barely noticeable. Distal subarticular tubercle on finger IV absent. Additional subarticular tubercles on fingers III and IV small and rounded, not exceeding phalangeal width. Subarticular tubercles on fingers I and II oval, protuberant and as wide as phalanges, corresponding to 2 and 1.4 times the width of the thenar tubercle, respectively. Supernumerary tubercles absent. Finger fringes and hand webbing absent. Tip of finger IV does not reach the distal subarticular tubercle of finger III when juxtaposed. Preaxial expansion of finger III, from the base to the tip of the finger. Finger I slightly longer than finger II. Relative length of fingers: IV < II < I < III. Discs of fingers II, III and IV slightly wider than the third phalange; disc of finger I of similar width to the distal phalange. Paired digital scutes present.

Thigh and tibia lengths similar, each 48% of body length. Foot length 88% of tibia length. Tarsal keel conspicuous, curved, straightening until almost forming a line towards, but not reaching, the inner metatarsal tubercle. Inner metatarsal tubercle protuberant and elliptical. Outer metatarsal tubercle small and rounded, protuberant, four times the diameter of the inner metatarsal tubercle. Metatarsal fold absent. Fringes on toes absent. Basal webbing present only between toes III and IV. Subarticular tubercle on toe I oval and protuberant. Subarticular tubercles on toes II–V rounded and barely evident; one subarticular tubercle on toe II, two on toes III and V, and three on toe IV. Disc of toe I same width as phalanx, tip rounded. Discs of toes II–V expanded laterally, tips rounded. Paired digital scutes present.

Skin on dorsum granular. Skin on throat, chest, belly and ventral surfaces of limbs smooth.

In preservative, dorsum of the body cream, with elevated concentration of dark granules and melanophores extending from the interorbital region to the urostyle (Fig. 3A). Dorsolateral stripe present. Dorsal region of arms, forearms, thighs and paracloacal marks cream (Fig. 3A). Tibia with dark brown patches of varied sizes. A dark brown lateral stripe surrounds the whole body, but it is narrower in the loreal region and of constant width in the lateral part of the body. Anterior and posterior regions of the thigh dark brown. Ventrolateral stripe present but barely noticeable and composed of irregular small whitish patches (Fig. 3B). Belly, chest, forearms and throat cream; melanophores grouped in the anterior portion of the throat. Ventral surface of arms, thighs and tibia cream, with melanophores present in the distal region of the thighs and peripheral regions of the tibia (Fig. 3C). Ventral surfaces of the hands and feet dark brown (Figs. 4A and 5B).

**Variation.** On average, females are slightly larger (SVL 16.7 mm ± 0.7, 16.0–17.4; *n* = 3) than males (SVL 15.4 mm ± 0.3, 14.9–16.2; *n* = 10) (Table 3). However, the small number of females impedes statistical tests to verify sexual dimorphism. Females differ from males by having thinner phalanges on finger III. In both males and females, the central region of the dorsum varies in the concentration of dark granules and melanophores: five specimens show a dense concentration of dark granules (thus resembling the holotype), whereas in six other specimens such a concentration is less evident. The dorsolateral stripe varies in width and visibility; in both males and females, it is absent or barely noticeable in life but more evident in preservative. The ventrolateral stripe is conspicuous in living specimens but almost invisible to the naked eye in preserved specimens, being formed by whitish patches with no melanophores. The anterior region of the throat has a variable amount of melanophores in both males and females. The tibia shows dark patches, which form a transverse line visible in five specimens but not in six others. The tarsal keel varies in size, shape and length; it reaches the external edge of the inner metatarsal tubercle in only one specimen, INPAH 41341.

**Table 3:** Morphometric measurements (in millimeters) of *Allobates velocicantus* sp. nov.

Characters	Holotype	Males (*n* = 10)	Females (*n* = 3)
SVL	15.3	15.4 ± 0.3 (14.9–16.2)	16.7 ± 0.7 (16.0–17.4)
IO	4.3	4.3 ± 0.2 (4.0–4.6)	4.6 ± 0.2 (4.4–4.8)
HW	5.3	5.2 ± 0.2 (5.0–5.5)	5.5 ± 0.2 (5.4–5.7)
HL	4.6	4.9 ± 0.2 (4.5–5.1)	5.2 ± 0.1 (5.1–5.3)
IN	2.3	2.2 ± 0.1 (2.2–2.4)	2.4 ± 0.1 (2.5–2.5)
LL	7.3	7.1 ± 0.2 (6.7–7.5)	7.4 ± 0.5 (7.1–8.0)
TL	7.4	7.4 ± 0.3 (6.6–7.7)	7.7 ± 0.4 (7.4–8.2)
FL	6.5	6.8 ± 0.3 (6.3–7.2)	7.1 ± 0.1 (7.0–7.2)
UAL	4.0	4.1 ± 0.2 (3.7–4.4)	4.4 ± 0.2 (4.2–4.6)
FAL	3.4	3.2 ± 0.2 (2.9–3.6)	3.3 ± 0.2 (3.2–3.5)
HANDI	3.0	3.2 ± 0.2 (2.9–3.7)	3.5 ± 0.4 (3.1–3.9)
HANDII	2.9	2.9 ± 0.2 (2.8–3.4)	3.2 ± 0.2 (3.0–3.3)
HANDIII	4.1	4.0 ± 0.2 (3.8–4.3)	4.2 ± 0.3 (4.1–4.5)
HANDIV	2.6	2.5 ± 0.2 (2.3–3.0)	2.7 ± 0.1 (2.6–2.8)
WFD	0.7	0.6 ± 0.1 (0.6–0.8)	0.7 ± 0.1 (0.6–0.8)
WPF	0.5	0.5 ± 0.1 (0.4–0.6)	0.4 ± 0.1 (0.4–0.5)
DPT	0.6	0.6 ± 0.0 (0.6–0.6)	0.7 ± 0.1 (0.6–0.8)
WTT	0.3	0.3 ± 0.1 (0.2–0.4)	0.3 ± 0.0 (0.3–0.4)
WTD	0.7	0.8 ± 0.1 (0.6–0.9)	0.8 ± 0.0 (0.8–0.9)
TYM	1.1	1.1 ± 0.1 (1.0–1.2)	1.0 ± 0.1 (1.0–1.1)
EL	2.3	2.3 ± 0.1 (2.2–2.5)	2.4 ± 0.1 (2.4–2.5)
EN	1.4	1.6 ± 0.1 (1.5–1.7)	1.7 ± 0.0 (1.7–1.7)
SL	2.2	2.5 ± 0.1 (2.4–2.8)	2.8 ± 0.1 (2.8–2.9)
FL/TL	0.9	0.9 ± 0.1 (0.8–1.1)	0.9 ± 0.0 (0.9–1.0)
EN/EL	0.6	0.7 ± 0.0 (0.6–0.7)	0.7 ± 0.0 (0.7–0.7)
HL/SVL	0.3	0.3 ± 0.0 (0.3–0.3)	0.3 ± 0.0 (0.3–0.3)
HW/SVL	0.4	0.3 ± 0.0 (0.3–0.4)	0.3 ± 0.0 (0.3–0.3)
HL/HW	0.9	0.9 ± 0.1 (0.9–1.0)	1.0 ± 0.0 (0.9–1.0)
FAL/SVL	0.2	0.2 ± 0.0 (0.2–0.2)	0.2 ± 0.2 (0.2–0.2)
TL/SVL	0.5	0.5 ± 0.0 (0.4–0.5)	0.5 ± 0.0 (0.5–0.5)

**Note:**

Values are presented as the mean ± standard deviation, with the range in parentheses.

In life, dorsum brown with dark brown granules concentrated medially. Dorsal surface of arms and forearms orange brown. Thighs light brown dorsally but dark brown at anterior and posterior ends. Supraorbital region light brown. Dark brown lateral stripe of constant width, narrower in the loreal region and wider in the flanks; small whitish dots inside the brown lateral line at the inguinal region. Dorsolateral stripe, when present, light brown. An irregular ventrolateral stripe is iridescent white. It is formed by dots in some specimens; in others, it is well defined and complete or incomplete (Figs. 6B, 6E, 6H and 6K). Ventrolateral surfaces of thighs and arms, as well as the flanks, are translucent beige. Belly white in males and females; chest white in females and translucent in males. Throat of females translucent with the edges of the jaw yellowish. In males, the vocal sac, when deflated, is whitish centrally and yellow peripherally. When inflated, it is mostly white with translucent yellow edges. Iris metallic bronze; pupils black, horizontal semielliptical.

**Comparisons with other species.** *Allobates velocicantus* sp. nov. easily differs from *A. femoralis* and *A. hodli* by the absence of bright yellow, orange or red marks on thighs (present). Cryptically colored species of *Allobates* show either of two striking dorsal color patterns, one with and the other without large dark patches or marks, a characteristic that helps to quickly differentiate species. *Allobates velocicantus* sp. nov. lacks a dark hourglass-shaped mark on the dorsum and is thus easily distinguished from *A. brunneus*, *A. carajas, A. crombiei, A. flaviventris*, *A. gasconi*, *A. magnussoni*, *A. ornatus*, *A. pacaas*, *A. tapajos*, and *A. trilineatus* (all of which have dark patches or marks on the dorsum with rhomboid-, diamond-, hourglass- or triangle-shaped patterns).

Among *Allobates* species that lack dark patches or marks on the dorsum, throat color is a reliable characteristic for differentiating species. Males of *Allobates velocicantus* sp. nov. has a white throat with yellow edges, which distinguishes it from males of *A. bacurau* (light to dark gray), *A. fuscellus* (dark to solid dark), *A. juami* (pinkish to translucent), *A. masniger* (dark gray), *A. melanolaemus* (black), *A. nidicola* (dark gray), *A. nunciatus* (violaceous), *A. paleovarzensis* (grayish-violet) and *A. vanzolinius* (light to dark gray).

*Allobates velocicantus* sp. nov. could be mistaken for *A. caeruleodactylus*, *A*. *conspicuus, A. grillisimilis, A. subfolionidificans* or *A. tinae*, which have similar ventral color patterns in preservative and adult males of similar size (SVL). However, *Allobates velocicantus* sp. nov. differs from *A*. *caeruleodactylus* by lacking basal webbing between fingers II–III and by scutes of fingers white (basal webbing between fingers II–III present, scutes of fingers blue); from *A. conspicuus* by having finger I slightly longer than finger II and by lacking both fringes on toes and transverse bars on thighs (finger I considerably longer than finger II, fringes on toes present and transverse bars on thighs present); from *A. grillisimilis* by the absence of a conspicuous transverse bar on legs and arms, finger III with phalanges of uniform width, edges of the throat yellow with center whitish (varying number of transverse bars on legs and arms present, finger III with phalanges of non-uniform width, and throat translucent white); and from *A. subfolionidificans* by having a white belly and a whitish throat with yellow edges, a ventrolateral stripe, and by lacking conspicuous transverse bars on the thighs (belly and throat opaque white, ventrolateral stripe absent, and transverse bars on thighs present).

**Figure 7:**
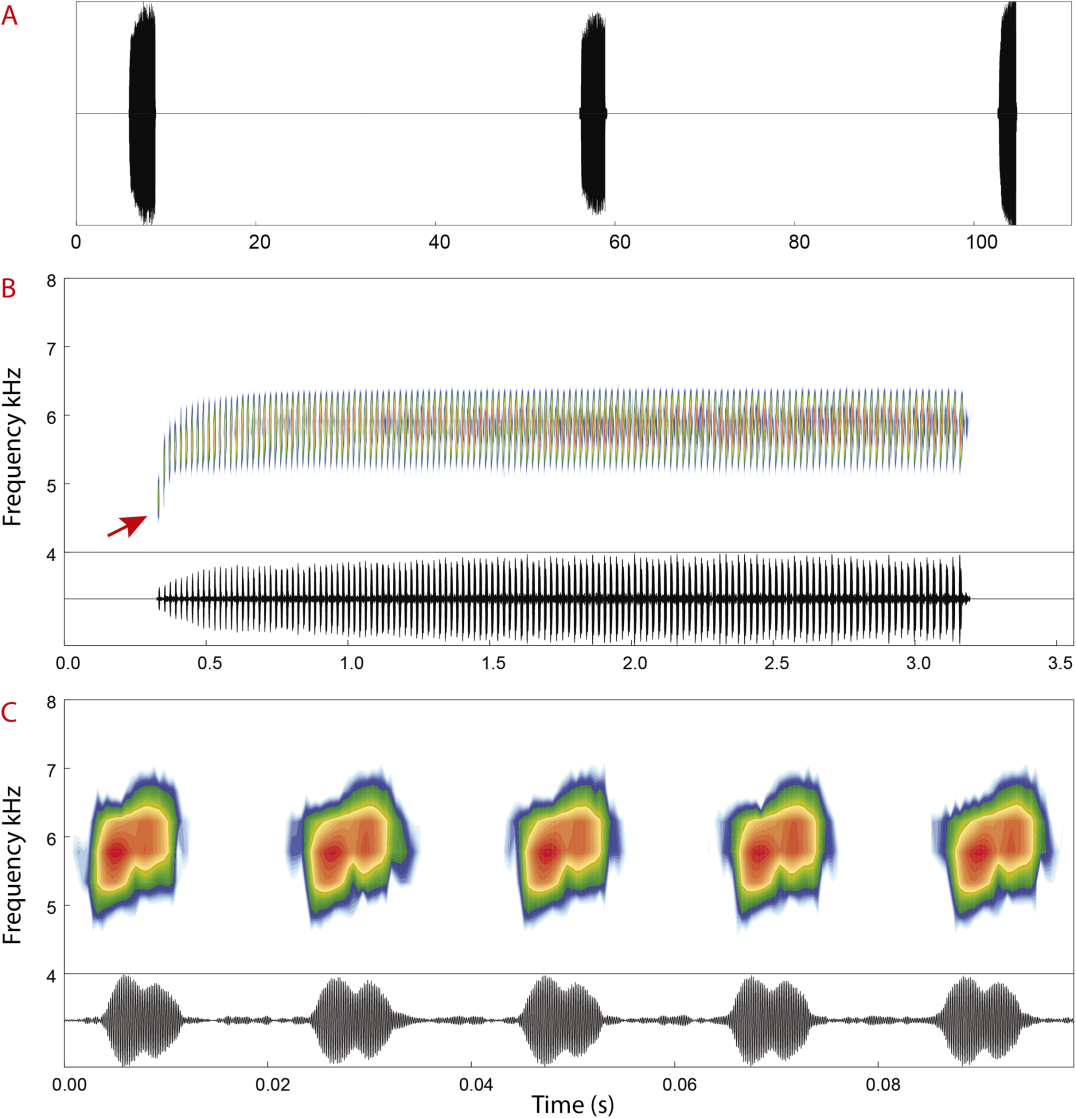
Advertisement call of *Allobates velocicantus* sp. nov. (A) Oscillogram showing three calls. (B) Spectrogram and oscillogram of a call composed of 137 pulsed notes. (C) Detailed view of five pulsed notes of the call depicted in (B). The red arrow denotes the first note, which has a lower dominant frequency (4,737 Hz) than later notes. Recorded male: INPAH 41346. Air temperature: 24.7 °C.

**Advertisement call.** Spectral and temporal parameters of eight recorded males are presented in Table 4. Advertisement calls of *Allobates velocicantus* sp. nov. (Fig. 7) comprise trills of 66–138 (121 ± 15) pulsed notes emitted at regular intervals, with a call duration of 1.87–2.89 s (2.49 ± 0.22). Inter-call intervals vary from 31.8 to 107.2 s (58.3 ± 16.6). Notes are emitted through multiple exhalations, are always composed of two pulses and have an average note duration of 8.7 ms ± 1.9 (5–13). Inter-note interval has an average duration of 13 ms ± 2 (10–18). The average dominant frequency is 5,831 Hz ± 168 (5,512–6,158). The dominant frequency of the first note of each call is considerably lower than that of other notes (Fig. 7B), varying from 4,263 to 5,900 Hz (5,133 Hz ± 386).

**Table 4:** Temporal and spectral characterization of advertisement calls of *Allobates velocicantus* sp. nov.

Voucher	SVL (mm)	°C	AC	CD (s)	ICI (s)	NNC	ND (ms)	INI (ms)	DF (Hz)	FNDF (Hz)	RR
INPAH 41338	16.2	26.0	5	2.1	57.3	121	8.9	12.0	5,551	5,108	56.8
INPAH 41339	15.5	24.7	3	2.4	48.9	130	9.6	13.0	5,756	4,809	53.9
INPAH 41341	15.4	24.7	3	2.2	78.5	119	9.6	10.0	5,835	4,888	53.8
INPAH 41342	15.3	26.0	2	2.6	45.9	126	8.0	12.0	5,871	5,369	49.0
INPAH 41343	15.5	26.0	1	2.6	71.9	133	9.4	11.0	5,900	5,060	51.5
INPAH 41346	15.6	24.7	4	2.4	53.2	123	8.2	12.0	5,922	4,802	50.4
INPAH 41348	14.9	27.4	2	2.2	31.8	123	7.1	12.0	5,965	5,426	55.6
INPAH 41350	15.5	26.0	4	2.8	66.7	107	9.0	12.0	6,094	5,828	38.4
Mean	15.5	25.7	3	2.4	58.3	121	8.7	13.0	5,831	5,133	51.2
SD	0.4	0.9	1	0.3	16.6	15	1.9	2.0	168	386	5.8
Min	14.9	24.7	1	1.9	31.8	66	5.0	10.0	5,512	4,263	38.4
Max	16.2	27.4	5	2.9	107.2	138	13.0	18.0	6,158	5,900	56.8

**Note:**

Values for mean, standard deviation (SD), minimum (Min) and maximum (Max) are based on all analyzed calls. Additional abbreviations: AC, number of analyzed calls; CD, call duration; DF, dominant frequency; FNDF, dominant frequency of the first note; ICI, inter-call interval; INI, inter-note interval; ND, note duration; NNC, number of notes per call; RR, repetition rate.

**Bioacoustic comparisons.** *Allobates velocicantus* is easily differentiated from *A. caeruleodactylus*, *A. magnussoni*, *A. masniger*, *A. nidicola* and *A. subfolionidificans* by emitting trills with 66–138 pulsed notes (advertisement call composed by a single note emitted continuously or irregularly in all these species: Lima & Caldwell, 2001; Caldwell & Lima, 2003; Lima, Simões & Kaefer, 2014; Lima, Sanchez & Souza, 2007; Tsuji-Nishikido et al., 2012).

The advertisement call of *A. velocicantus* sp. nov. has only one type of temporal arrangement of notes (trills), which distinguishes it from the calls of *A. brunneus*, *A. carajas*, *A. flaviventris* and *A. tapajos* (each of these species has two types of temporal arrangement of notes: notes emitted continuously, or in trills of unpulsed notes). In addition, by emitting trills with 66–138 pulsed notes, *A. velocicantus* sp. nov. differs from *A. brunneus* (trills of 6–11 notes; Lima, Caldwell & Strüssmann, 2009), *A. carajas* (trills of 4–22 notes; Simões, Rojas & Lima, 2019), *A. flaviventris* (trills of 2–10 notes; Melo-Sampaio, Souza & Peloso, 2013) and *A. tapajos* (trills of 10–14 notes; Lima, Simões & Kaefer, 2015).

Since the advertisement call of *A. velocicantus* sp. nov. has a maximum duration of 2.9 s and is composed of trills with as many as 138 notes and inter-note intervals of 10–18 ms, the new species can be differentiated from *A. bacurau* (maximum call duration 11.1 s, maximum notes 81, inter-note interval 71–129 ms; Simões, 2016), *A. crombiei* (maximum call duration 4.5 s, maximum notes 59, inter-note interval 45–69 ms; Lima, Erdtmann & Amézquita, 2012), *A. femoralis* (maximum call duration 0.7 s, maximum notes 6, inter-note interval 51–140 ms; Moraes, Pavan & Lima, 2019), *A. granti* (maximum call duration 4.5 s, maximum notes 17, inter-note interval 110–250 ms; Kok et al., 2006), *A. hodli* (maximum call duration 0.2 s, maximum notes 2, inter-note interval 62–99 ms; Simões, Lima & Farias, 2010), *A. insperatus* (maximum call duration 3.7 s, maximum notes 45, inter-note interval 42–85 ms; Simões et al., 2018), *A. juami* (maximum call duration 5.1 s, maximum notes 73, inter-note interval 20–50 ms; Simões et al., 2018), *A. nunciatus* (maximum call duration 357 ms, maximum notes 10, inter-note interval 29–85 ms; Moraes, Pavan & Lima, 2019), *A. paleovarzensis* (maximum call duration 3.0 s, maximum notes 21, inter-note interval 65–266 ms; Lima et al., 2010), *A. tinae* (maximum call duration 3.7 s, maximum notes 13, inter-note interval 100–410 ms; Lima, Ferrão & Silva, in press) and *A. trilineatus* (maximum call duration 1.6 s, maximum notes 13, inter-note interval 71–88 ms; Grant & Rodríguez, 2001).

**Color of freshly laid eggs and larvae description.** Freshly laid eggs of *A. velocicantus* sp. nov. have dark gray animal pole covering approximately two thirds of the animal hemisphere. The dark gray animal pole shows a well-delimited edge above the whitish equatorial portion. The vegetal pole is completely whitish. Eggs are deposited in a cloudy jelly.

**Figure 8:**
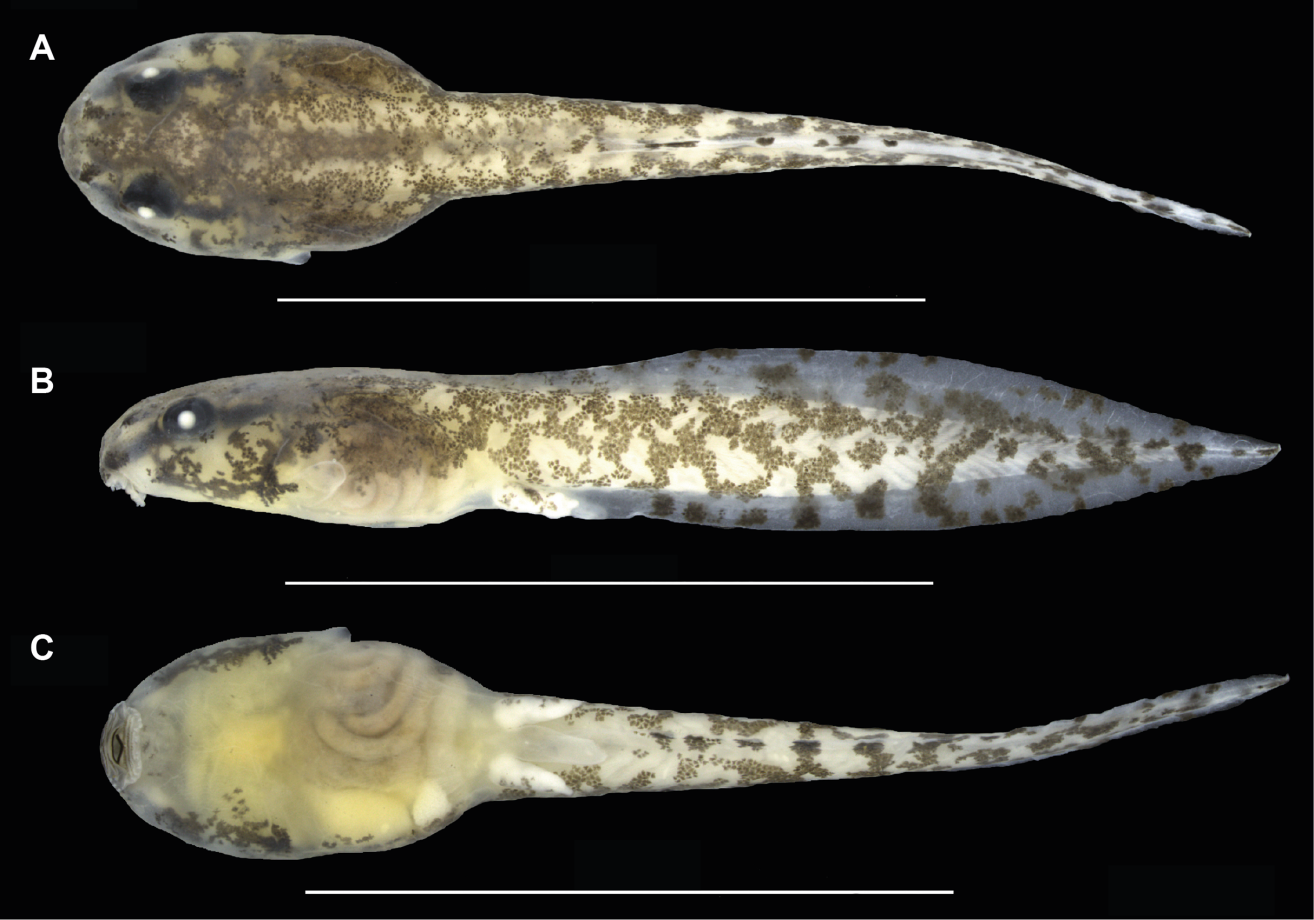
Preserved tadpole of *Allobates velocicantus* sp. nov. (INPAH 41351) at Gosner stage 37. (A) Dorsal, (B) lateral and (C) ventral views. Scale bars: 10 mm. Photographs by Jeni Lima Magnusson.

**Figure 9:**
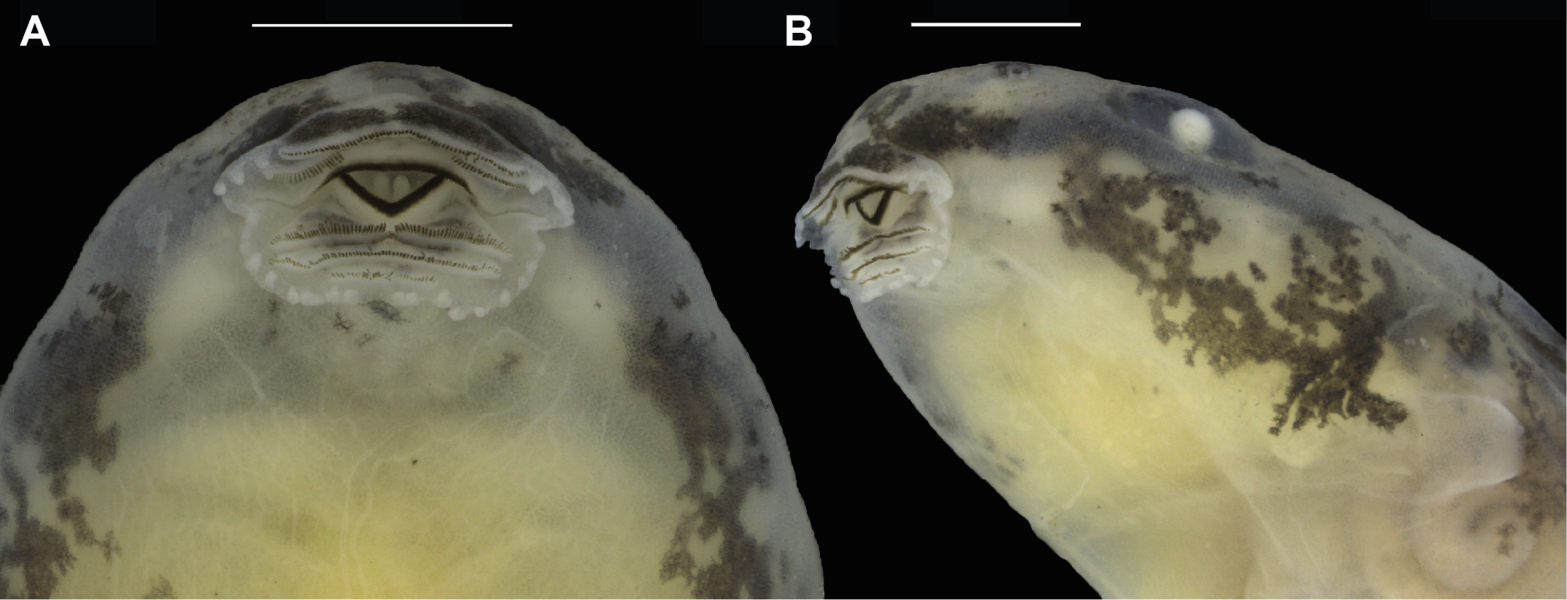
Oral disc of a preserved tadpole of *Allobates velocicantus* sp. nov. (INPAH 41351). (A) Ventral and (B) ventrolateral views. Scale bars: 1 mm. Photographs by Jeni Lima Magnusson.

Morphometric measurements of tadpoles are presented in Table 5. See Figs. 8 and 9 for body form and oral disc. Descriptions of quantitative characters are based on tadpoles at Gosner stage 34 (*n* = 5). Body ovoid, rounded anteriorly and posteriorly in dorsal view, flattened in lateral view (BH/BW = 0.7–0.8). Body length and tail length 30.7–33.0% and 66.9–69.3% of TL, respectively; HWLE 81.5–90.9% of BW; snout rounded in lateral and dorsal views; END 60.0–76.5% of ED; eyes dorsal and directed laterally; IOD 54.8–58.6% of HWLE. Small nares located dorsolaterally and directed anterolaterally, visible in dorsal and lateral views; internarial distance 40.0–45.2% of HWLE. Fleshy ring on inner margin of nostrils rounded, not ornamented. Spiracle single, sinistral, tubular, 0.6–0.9 mm long, attached to body ventrolaterally, slightly below mid-body length. Gut coiled and visible through the skin to the naked eye, with its axis directed to the left side of the body. Vent tube dextral, 1.4–2.2 mm long. Maximum tail height 2.5–2.9 mm. Dorsal fin emerges after 2.0–2.5 mm from the limit between tail and body; dorsal edge shallow and straight anteriorly (along approximately 10% of its length) but deeper posteriorly, reaching maximum height at two thirds of the tail length. Dorsal fin slightly deeper than ventral fin. Ventral fin does not exceed body height. Tail tip slightly acuminate. Caudal musculature in dorsal view attains 47.4–51.5% of body width. In lateral view it reaches 54.8–75.0% body height. Oral disc located anteroventrally, emarginated laterally (Fig. 8); width of oral disc 1.4–1.6 mm, corresponding to approximately 41.6–45.5% of body width at the level of spiracle. Anterior labium with 3–4 short, pyramidal, rounded papillae distributed in a single row on each lateral margin. Posterior labium with a single row of 16–21 marginal papillae of similar size and shape to those on anterior labium. Submarginal papillae absent. Upper jaw sheath arc-shaped, longer than lower jaw sheath, with no medial notch. Lower jaw sheath V-shaped, slightly wider than upper jaw sheath. Edge of upper and lower jaw sheaths serrated; serrations extend along the entire length of each sheath. Labial keratodont row formula (LKRF) 2(2)/3(1). Tooth row A-1 complete, 1.1 ± 0.2 mm long; A-2 is 1.1 ± 0.1 mm long and interrupted by a medial gap of 0.5 ± 0.0 mm. Tooth row P-1 is 1.0 ± 0.1 mm long with a small medial gap. Tooth row P-2 is complete and 0.9 ± 0.1 mm long. Tooth row P-3 is complete but shorter than P-1 and P-2, 0.5 ± 0.1 mm long.

**Table 5:** Morphometric measurements (in millimeters) of 14 tadpoles of *Allobates velocicantus* sp. nov., Gosner stages 27–37, collected in the municipality of Mâncio Lima, state of Acre, Brazil.

	Stage 27 (*n* = 1)	Stage 31 (*n* = 1)	Stage 32 (*n* = 3)	Stage 33 (*n* = 2)	Stage 34 (*n* = 5)	Stage 36 (*n* = 1)	Stage 37 (*n* = 1)
TL	13.6	16.0	16.5 ± 0.6 (15.8–16.9)	17.5–17.7	17.2 ± 1.07 (15.7–18.5)	19.3	19.6
BL	4.5	5.2	5.4 ± 0.2 (5.1–5.5)	5.8–6.0	5.6 ± 0.3 (5.2–6.1)	6.3	6.6
TAL	9.1	10.8	11.1 ± 0.4 (10.7–11.5)	11.5–11.9	11.6 ± 0.8 (10.5–12.4)	13.0	13
BW	2.9	3.5	3.4 ± 0.4 (3.0–3.7)	3.6 –3.7	3.4 ± 0.2 (3.2–3.8)	3.7	4.1
BH	1.9	2.6	2.4 ± 0.4 (2.0–2.7)	2.7–2.9	2.5 ± 0.3 (2.3–3.1)	2.8	3.1
HWLE	2.3	3.0	2.8 ± 0.1 (2.7–2.9)	2.8–3.2	2.9 ± 0.1 (2.8–3.1)	3.2	3.2
TMW	1.1	1.4	1.5 ± 0.1 (1.4–1.6)	1.7–1.7	1.7 ± 0.1 (1.6–1.8)	1.5	1.9
MTH	2.0	2.4	2.5 ± 0.1 (2.4–2.5)	2.5–2.7	2.7 ± 0.2 (2.5–2.9)	2.9	2.8
TMH	1.1	1.5	1.5 ± 0.1 (1.4–1.6)	1.7–1.7	1.7 ± 0.1 (1.6–1.8)	1.7	1.8
IOD	1.3	1.5	1.6 ± 0.1 (1.5–1.6)	1.6–1.7	1.7 ± 0.1 (1.6–1.7)	1.7	1.9
IND	0.9	1.3	1.2 ± 0.1 (1.1–1.3)	1.4–1.4	1.3 ± 0.1 (1.2–1.4)	1.5	1.6
END	0.5	0.5	0.6 ± 0.0 (0.6–0.6)	0.7–0.7	0.6 ± 0.0 (0.6–0.7)	0.6	0.8
NSD	0.4	0.3	0.4 ± 0.0 (0.4–0.5)	0.5–0.5	0.4 ± 0.1 (0.3–0.5)	0.3	0.5
ED	0.8	0.9	0.8 ± 0.1 (0.8–0.9)	1.0–1.0	0.9 ± 0.1 (0.9–1.0)	1.0	1.1
SS	2.7	3.0	3.0 ± 0.0 (2.7–3.4)	3.6–3.8	3.4 ± 0.3 (3.1–3.9)	3.1	3.9
VTL	1.1	1.3	1.5 ± 0.1 (1.4–1.6)	1.6–1.9	1.7 ± 0.3 (1.4–2.16)	2.0	1.7
STL	0.3	0.5	0.7 ± 0.1 (0.6–0.9)	0.8–0.8	0.7 ± 0.1 (0.6–0.9)	0.6	0.8
ODW	1.2	1.5	1.4 ± 0.1 (1.3–1.4)	1.4–1.5	1.5 ± 0.1 (1.4–1.6)	1.6	1.7
PL	0.4	0.4	0.4 ± 0.0 (0.4–0.4)	0.3–0.5	0.4 ± 0.1 (0.3–0.5)	0.6	0.6
AL	0.5	0.5	0.5 ± 0.1 (0.4–0.5)	0.5–0.5	0.5 ± 0.0 (0.4–0.5)	0.5	0.5
GAP	0.4	0.4	0.4 ± 0.0 (0.4–0.4)	0.4–0.4	0.4 ± 0.1 (0.2–0.5)	0.4	0.6
A1	0.9	1.0	0.9 ± 0.0 (0.9–0.9)	1.0–1.0	1.0 ± 0.2 (0.7–1.3)	NA	NA
A2	0.7	1.1	1.0 ± 0.0 (1.0–1.0)	1.1–1.2	0.9 ± 0.4 (0.3–1.2)	1.2	1.1
P1	0.8	1.0	1.0 ± 0.0 (0.9–1.0)	0.9–1.1	1.0 ± 0.1 (1.0–1.1)	1.1	1.3
P2	0.7	0.9	0.9 ± 0.0 (0.8–0.9)	0.8–1.1	0.9 ± 0.1 (0.9–1.1)	1.0	1.2
P3	0.5	0.6	0.5 ± 0.1 (0.3–0.6)	0.6–0.6	0.5 ± 0.1 (0.4–0.6)	0.4	0.7
UJW	NA	0.1	0.1 ± 0.0 (0.1–0.1)	0.1–0.1	0.1 ± 0.0 (0.1–0.1)	0.1	0.2
UJL	0.7	0.7	0.7 ± 0.0 (0.7–0.7)	0.7–0.7	0.8 ± 0.0 (0.7–0.8)	0.8	0.8

**Note:**

For stages with more than two tadpoles, values represent the mean ± standard deviation (minimum–maximum). For stages with only two tadpoles, values represent the minimum and maximum. Abbreviations: n, sampling size; NA, not measured. See the main text for abbreviations of morphometric measurements.

In preservative, dorsal, lateral and anteroventral surfaces of body cream, covered with aggregations of brown melanophores and blotches (Fig. 8). Tail fins transparent cream with brown blotches. Higher concentration of reticular-shaped blotches over tail muscle. Ventral region translucent, with some melanophores at the peripheral region and internal organs visible through the skin.

**Comparisons with tadpoles of other species**. Tadpoles of *Allobates velocicantus* sp. nov. have short, rounded and pyramidal papillae on each side of the labium. There are 3 or 4 papillae on the anterior labium and from 16 to 21 on the posterior labium. Hence, this species is distinct from congeners that have more-elongate papillae on the posterior labium—*A. caeruleodactylus* (6 long papillae), *A. grillisimilis* (approximately 8 very long papillae), *A. subfolionidificans* (40 papillae on the posterior labium, which are longer than those on the anterior labium) and *A. tapajos* (8–10 long papillae)—and from those that have a larger number of short papillae on the anterior labium: *A. brunneus* (5 papillae), *A. carajas* (5–8 papillae), *A*. *magnussoni* (12–13 papillae) and *A. nunciatus* (8–9 papillae).

Tadpoles of *Allobates velocicantus* sp. nov. have an arrangement of posterior tooth rows with P1 > P2 > P3 and P3 equivalent to around half of P2, thus being distinct from tadpoles of *A. palevarzensis* (P2 > P1 > P3) and *A. hodli* (P2 = P1 = P3). The LKRF in tadpoles of *A. velocicantus* sp. nov. is 2(2)/3(1), which differs from the LKRF in *A. femoralis* (2(2)/3).

**Figure 10:**
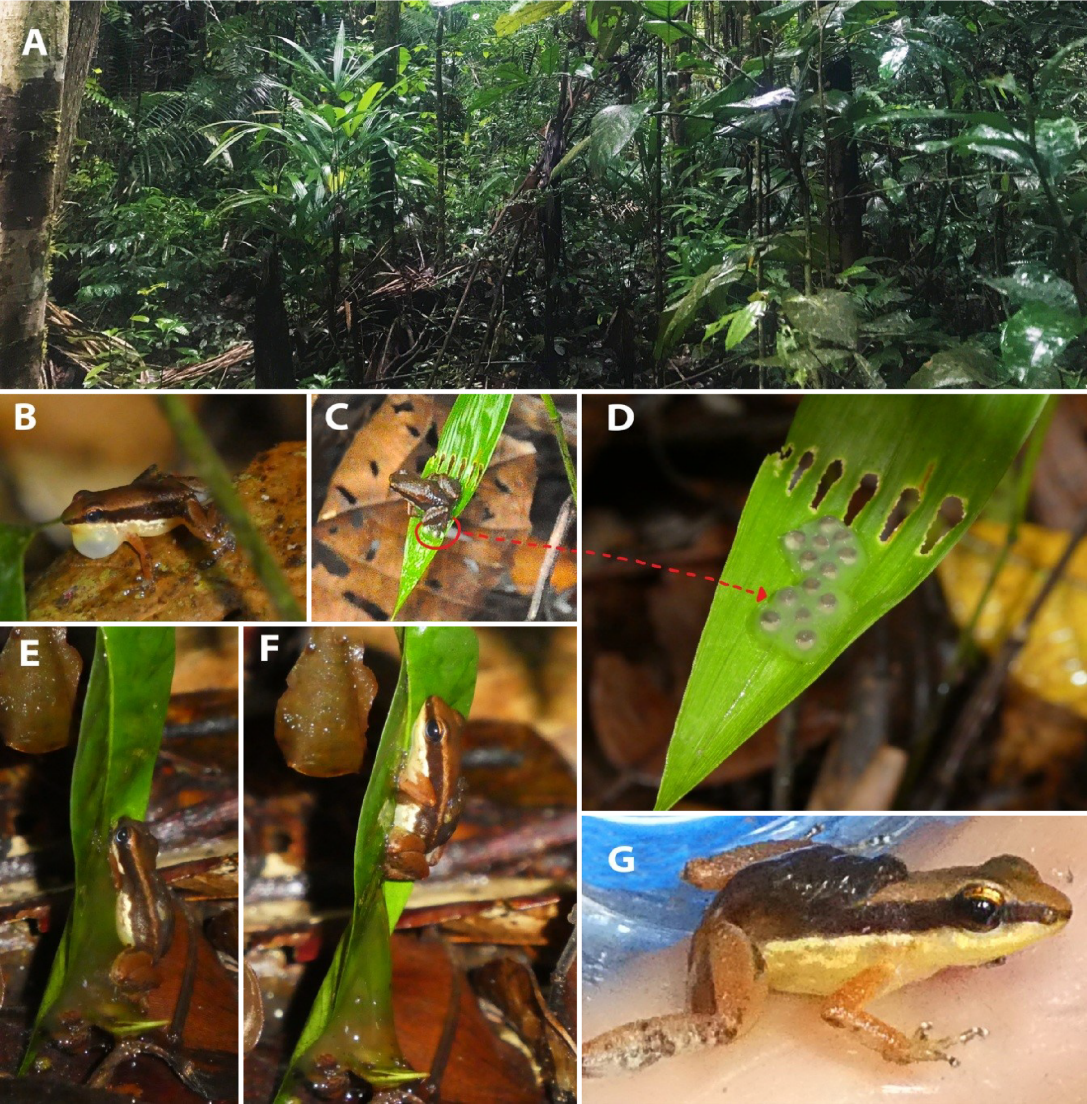
Habitat and breeding behavior of *Allobates velocicantus* sp. nov. (A) Understory of the lowland ombrophilous open forest at the type locality, municipality of Mâncio Lima, state of Acre, Brazil. (B) A calling male (INPAH 41343). (C) A female ovipositing (uncollected). (D) Egg clutch containing 13 eggs with animal pole covered by melanin. (E and F) Male collecting tadpoles to transport to a pond (INPAH 41347). (G) Male with tadpoles on his back (INPAH 41347). In both clutches, eggs were deposited on the adaxial surfaces of green leaves. Photographs by Jesus R. D. Souza.

**Natural history notes.** *Allobates velocicantus* sp. nov. inhabits the litterfall of primary and secondary lowland ombrophilous open forest (Fig. 10A). Populations of this species were found in *terra firme* forest either close to or distant from small forest streams. The new species uses leaves of small shrubs in the forest understory as egg deposition sites; two clutches (nine tadpoles and 13 eggs) were found in this situation. On 13–15 February 2019, during the middle of the rainy season, males were found calling between 8:00 and 18:00 h, and always perched between 10 and 30 cm above the ground. A pair of adults was observed courting: the male was leading the female to an egg deposition site, and he emitted a courtship call while both frogs were moving. As the male started emitting another advertisement call, the female stopped following. Arriving at the egg deposition site, the male jumped on to a leaf (adaxial surface), which was located around 20 cm above the ground, and continued to emit advertisement calls interspersed with courtship calls. After a few moments, the female jumped to the leaf with the male and approached him, initially touching snouts before turning her back to him. The male then jumped onto the female’s dorsum, while also sliding its hand to her head. The male remained in that position—cephalic amplexus—for 2 min before jumping from the leaf. The female remained in place for a few moments but then began moving, turning clockwise 30°, stopping, and repeating that pattern several times, always turning clockwise. The length of time the female stopped between movements ranged from 2 to 10 min, while she deposited eggs (Fig. 10C). Overall, egg deposition lasted 40 min before the female abandoned the leaf, leaving behind a clutch of 13 eggs. Each egg had a distinctly pigmented animal pole and was encased within cloudy jelly (Fig. 10D). The clutch was collected immediately after the female abandoned it, the embryos maintained alive through hatching, and the resulting tadpoles reared to Gosner stages 27–37 before being sacrificed and preserved.

The second egg clutch had nine tadpoles at aquatic-transport stage. The male (INPAH 41347) jumped onto the egg clutch and made circular movements inside it, at which time the tadpoles began to wriggle towards his dorsum, eventually climbing onto it (Figs. 10E–10G). This event lasted for 5 min.

## Discussion

*Allobates velocicantus* is the twenty-sixth species of *Allobates* described from Brazil and the twenty-third species from Brazilian Amazonia. The species is distributed in southwest Amazonia, a region of high species richness for several taxonomic groups (Jenkins, Pimm & Joppa, 2013), including anurans (Souza, 2009; Bernarde, Machado & Turci, 2011). The type locality is located in the Area of Relevant Ecological Interest Japiim Pentecostes, a conservation unit intended for sustainable use. Yet, the conservation of *A. velocicantus*, and of regional biodiversity in general, is not guaranteed. Deforestation in this part of Amazonia, including in conservation units, has increased with the expansion of illegal logging, cattle ranching, and agriculture (Kröger, 2020; Montibeller et al., 2020). Moreover, deforestation and degradation rates in Amazonia are increasing substantially due to the anti-environmental agenda of the current Brazilian government (Ferrante & Fearnside, 2019; Kröger, 2020).

The high note-repetition rate call of *Allobates velocicantus* is a remarkable feature. Four species of *Allobates* emit calls with similar structure: *A. bacurau*, *A. crombiei*, *A. juami* and *A. insperatus*. However, the note-repetition rate in *A. velocicantus* is strikingly higher than the rates of these other species. Furthermore, the advertisement call of *A. velocicantus* has the highest number of notes among all species of *Allobates* with documented calls. These characters make the species unique and easily distinguished from congeners. While most species of *Allobates* share a conserved external morphology, vocalizations are highly variable and species-specific. They represent essential characters for use in the formal diagnosis and description of new taxa, as well as in future species discovery.

Information on egg coloration in *Allobates* is scarce except for species recently described or redescribed (Lima, Sanchez & Souza, 2007; Lima, Caldwell & Strüssmann, 2009; Lima et al., 2010; Lima, Simões & Kaefer, 2014, 2015; Moraes, Pavan & Lima, 2019; Simões et al., 2013; Simões, Rojas & Lima, 2019). *Allobates velocicantus* oviposits on green leaves of small shrubs. Currently, only two other species in Brazilian Amazonia use this breeding strategy exclusively: *A. subfolionidificans* and *A. carajas*. Unlike in *A. velocicantus*, however, the animal pole of eggs in these two species is white (Lima, Sanchez & Souza, 2007; Souza, Kaefer & Lima, 2017; Simões, Rojas & Lima, 2019). Despite both *A. velocicantus* and *A. subfolionidificans* inhabiting the open-canopy lowland forest with considerable incidence of sun rays above canopy, eggs of *A*. *subfolionidificans* are protected from direct solar radiation by being deposited on the abaxial surfaces of green or dry leafs of small shrubs (Lima, Sanchez & Souza, 2007; Souza, Kaefer & Lima, 2017), while *A. velocicantus* oviposits on the adaxial surface of green leaves of small shrubs. On the other hand, *A. carajas* oviposits white eggs on the adaxial surface of green leaves (Simões, Rojas & Lima, 2019), but eggs are protected by a dense-canopy forests with low levels of solar incidence on the lower understory vegetation (P.I. Simões, 2014, personal communication). Intense solar radiation may cause mortality and abnormal embryo development in anurans (Beudt, 1930; Gurdon, 1960; Blaustein et al., 1997), so the amount of melanin on eggs in these species might be an ecophysiological response to the solar radiation to which eggs are exposed. Differences in the amount of melanin on eggs were reported from other two species of the *A. tinae* species complex inhabiting forests with distinct canopy openness in the Brazilian Amazonia (Lima, Ferrão & Silva, in press). The color of jelly may have a protective function against solar radiation. Jelly of *A. velocicantus* is cloudy, while the jelly of *A*. *subfolionidificans* and *A. carajas* are translucent. The effect of solar radiation on eggs of *Allobates* with different concentrations of melanin and deposited within jellies of different colors needs physiological tests.

## Conclusion

*Allobates velocicantus* is differentiated from its congeners based on external morphology of adults and tadpoles, advertisement call and molecular analyses. The species represents an excellent model to study the ecological and physiological adaptations to solar radiation on eggs of *Allobates*. However, the conservation of *A. velocicantus* is threatened by the expansion of illegal logging, cattle ranching, and agriculture.

## Appendix I. Specimens Examined

***Allobates tinae***. Adults. Brazil: Rondônia: Porto Velho, west bank of upper Madeira River [INPAH 41012–21, 41029–36, 41041–44]; Amazonas: Boca do Acre (INPAH 40976, 41022, 41027, 41037, 41040).

***Allobates* aff. *tinae***. Adults. Brazil: Amazonas: Careiro, RAPELD M1 at km 32 of the Brazilian federal highway BR-319 [INPAH 41045–47, 41049–53, 41055–57, 41059, 41061, 41063, 41067–69].

***Allobates bacurau***. Adults. Brazil: Amazonas: Estrada do Miriti, Manicoré [INPAH 35398 (holotype); 35397, 35399–35409 (paratypes)].

***Allobates brunneus***. Adults. Brazil: Mato Grosso: NE of Chapada dos Guimarães [INPAH 10111–48 (topotypes)]. Tadpoles. Brazil: Mato Grosso: NE of Chapada dos Guimarães. INPAH 10025–10027, 10029–10030, 10032–10037, 10039, 10041, 10043, 10044 (topotypes).

***Allobates caeruleodactylus***. Adults. Brazil: Amazonas: km 12 on the road to Autazes [INPAH 7238 (holotype); 7229–7232, 7234–7237 (paratypes)]. Tadpoles. Brazil: Amazonas: km 12 on the road to Autazes (INPAH 8037–8046, 8085).

***Allobates crombiei***. Adults. Brazil: Pará: Cachoeira do Espelho [INPAH 30457–30477 (topotypes)].

***Allobates femoralis***. Adults. Brazil: Pará: Treviso (INPAH 11657–11671, 15232, 30769–30778); Pará: Itaituba (INPAH 26342–26354).

***Allobates fuscellus***. Adults. Brazil: Amazonas: Ipixuna: Penedo, east bank of Juruá river [INPAH 2531 (paratopotype); 2532 (holotype)]; Itamarati: Jainu, Juruá River [INPAH 3114, 3250, 3270, 3514 (paratypes)].

***Allobates gasconi***. Adults. Brazil: Amazonas: Itamarati: Jainu, west bank of Juruá River [INPAH 3082 (holotype); 3073, 3079, 3085, 3090, 3150, 3151, 3172, 3249, 3406, 3415, 3483, 3484, 3491, 3494, 3496, 3512, 3513 (paratypes)].

***Allobates grillisimilis***. Adults. Brazil: Amazonas: Borba [INPAH 30779 (holotype); 30780–30808 (paratopotypes)]; Nova Olinda do Norte [INPAH 30809–30823 (paratypes)].

***Allobates magnussoni***. Adults. Brazil: Pará: Parque Nacional da Amazônia [INPAH 32960 (holotype); 32961–32976, 32978– 32982 (paratopotypes)]; Treviso (INPAH 10105–10109, 33930–33934). Tadpoles. Brazil: Pará: Treviso (INPAH 10054, 10056, 10058, 10059, 10060).

***Allobates masniger***. Adults. Brazil: Amazonas: Borba (INPAH 28075, 28078, 28084, 28089, 28092, 28095, 28098, 28100, 28104, 28105, 28112, 28114, 28119); Pará: Parque Nacional da Amazônia [INPAH 28195–28217 (topotypes)]; Jacareacanga (INPAH 28053, 28070, 28077, 28082, 28093, 28094, 28099, 28103, 28107, 28110, 28111, 28113, 28115, 28118, 28120).

***Allobates nidicola***. Adults. Brazil: Amazonas: km 12 on road to Autazes [INPAH 8093 (holotype); INPAH 7253–7259, 7261, 7262, 8094 (paratypes); INPAH 28122, 28124, 28127, 28129, 28131, 28144, 28159, 28163, 28166, 28169, 28171, 28172, 28174, 28179, 28184, 28185 (topotypes)]. Tadpoles. (INPAH 8021–8033, 8137–8139).

***Allobates nunciatus***. Adults. Brazil: Pará: Itaituba [INPAH 40486 (holotype); INPAH 40305, 40307, 40475, 40480, 40485, 40489, 40324, 40476 (paratypes)]; Trairão [INPAH 40482, 40484, 40488 (paratypes)].

***Allobates paleovarzensis***. Adults. Brazil: Amazonas: Careiro da Várzea [INPAH 20904 (holotype); INPAH 20861–20903, 20905 (paratypes)].

***Allobates subfolionidificans***. Adults. Brazil: Acre: Parque Zoobotânico da Universidade Federal do Acre [INPAH 13760 (holotype); INPAH 11958–11974, 13749–13754, 13756–13759, 13761, 13762 (paratypes)]. Tadpoles. Brazil: Acre: Parque Zoobotânico da Universidade Federal do Acre (INPAH 14822, 14823).

***Allobates tapajos***. Adults. Brazil: Pará: Parque Nacional da Amazônia [INPAH 34402–34424 (paratypes); INPAH 34425 (holotype)]. Tadpoles. Brazil: Pará: Parque Nacional da Amazônia (Lots INPAH 34426, 34427).

***Allobates trilineatus***. Adults. Brazil: Acre: Parque Zoobotânico da Universidade Federal do Acre (INPAH 11958–11993).

***Allobates vanzolinius***. Adults. Brazil: Amazonas: Vai-Quem-Quer, Rio Juruá [INPAH 4896 (holotype); INPAH 4903, 4904, 4905, 4912 (paratypes)]; Jainu, Rio Juruá [INPAH 3381, 3413 (paratypes)].

## Supplemental Information

10.7717/peerj.9979/supp-1 Supplemental Information 1 Voucher, sampling locality, and GenBank accession number of samples used for the phylogenetic analyses.

10.7717/peerj.9979/supp-2 Supplemental Information 2 Morphometric measurements of the type series of *Allobates velocicantus* sp. nov. Morphometric abbreviations are described in the main text.

10.7717/peerj.9979/supp-3 Supplemental Information 3 16S sequences of the new Species. sequences MT446458 to MT446462 in fasta format.

## Additional Information and Declarations

### Field Study Permissions

The following information was supplied relating to field study approvals (i.e., approving body and any reference numbers): Specimens were collected under collection permit number 1337-1 provided by the Instituto Brasileiro do Meio Ambiente e dos Recursos Naturais Renováveis (IBAMA).

### Data Availability

The following information was supplied regarding data availability: Specimens are housed in the herpetological section of the Zoological Collection of the Instituto Nacional de Pesquisas da Amazônia (INPAH), Manaus, Amazonas, Brazil, under numbers INPAH 41339 to INPAH 41351. Sequences are available at GenBank under accession numbers MT446458 to MT446462. Morphometric measurements are provided in a Supplemental Table. Advertisement calls are housed at Fonoteca Neotropical Jacques Vielliard (FNJV 45469–79), UNICAMP, Campinas, Brazil. In addition, call recordings are available at Figshare: Souza, Jesus; Ferrão, Miquéias; Hanken, James; Lima, Albertina Pimentel (2020): Advertisement call of a new species of *Allobates* from Acre, Brazil. figshare. Media. DOI 10.6084/m9.figshare.12283973.v1.

### New Species Registration

The following information was supplied regarding the registration of a newly described species: Publication LSID: urn:lsid:zoobank.org:pub:B411120A-AB6C-4091-81D7-24D52B377BEE. *Allobates velocicantus* sp. nov. LSID: urn:lsid:zoobank.org:act:8EAF9324-F176-4A5F-8788-9ACE3E885173.
